# Study on Volatile Profiles, Polycyclic Aromatic Hydrocarbons, and Acrylamide Formed in Welsh Onion (*Allium fistulosum* L.) Fried in Vegetable Oils at Different Temperatures

**DOI:** 10.3390/foods11091335

**Published:** 2022-05-04

**Authors:** Hye-Min Kim, Min-Kyung Park, Soo-Jeong Mun, Mun-Yhung Jung, Sang-Mi Lee, Young-Suk Kim

**Affiliations:** 1 Department of Food Science and Biotechnology, Ewha Womans University, Seoul 03760, Korea; hmhm33@ewhain.net (H.-M.K.); carrot0412@gmail.com (M.-K.P.); msz4710@ewhain.net (S.-J.M.); 2 School of Food Science, Woosuk University, Samrea-up, Wanju-Kun 55338, Korea; munjung@woosuk.ac.kr; 3 Department of Food and Nutrition, Inha University, Incheon 22212, Korea; smlee21@inha.ac.kr

**Keywords:** Welsh onion, frying oil temperature, vegetable oil, volatile compounds, polycyclic aromatic hydrocarbons, acrylamide

## Abstract

Welsh onion (*Allium fistulosum* L.) is widely used in diverse Asian cuisines, especially in stir-fried and deep-fried foods. This study investigated the effects of different temperatures (140, 165, and 190 °C) and types of the vegetable frying oil (soybean, corn, canola, and palm oils) on the formation of volatile profiles and hazardous compounds [polycyclic aromatic hydrocarbons (PAHs) and acrylamide] in Welsh onion. Specific volatile chemical groups such as aldehydes, sulfur-containing compounds, and furans/furanones were major volatiles in Welsh onion fried (WOF). The composition of aldehydes and sulfur-containing compounds decreased, while those of furans/furanones increased when WOF samples were exposed to higher temperatures. At 190 °C, PAHs were detected at lower than the EU maximum tolerable limit (the sum of 4 PAHs, <10 µg/kg), and acrylamide was detected below 36.46 μg/kg. The integrated study of both the quality and safety properties can provide fundamental data for the industrial processing of WOF.

## 1. Introduction

Welsh onion (*Allium fistulosum* L.) belongs to the *Allium* genus in the Alliaceae family and is widely used to enhance the flavor of various dishes, in particular stir-fried and deep-fried foods in East Asia. When Welsh onion is heated with oil at high temperature, diverse organic compounds of the Welsh onion and the oil can react to produce volatile compounds that contribute to unique flavors, resulting in oil rich in a “vegetable” and “burnt” flavor.

Studies have investigated the volatile compounds generated from plants of the Allium genus, such as garlic, onion, shallot onion, and Welsh onion, during heating with oil. The flavors formed can be affected by various factors, including the frying temperature and oil type. Sun et al. [1] demonstrated that the flavor characteristics of garlic oil vary with the frying temperatures; for example, increasing the frying temperature resulted in the content of thioethers and heterocycles being higher than those of other volatile compounds, especially 2,6-dimethylpyrazine, dimethyl trisulfide, and diallyl disulfide. Another study investigated the formation of flavor characteristics in fried shallot prepared with different oils, such as rapeseed, peanut, soybean, and sunflower oils [2]. In the results, 2-ethyl-3,5-dimethyl-pyrazine was related to a shallot-scent flavor. Zhang et al. [3] explained that certain interactions, such as those between carbohydrates, proteins, and fats, and the thermal degradation of sugars, amino acids, and fats could lead to the forming of flavor volatile compounds in the oil used for Welsh onion fried (WOF). Furans/furanones, sulfur-containing compounds, aldehydes, and alcohols were found as the most important contributors to the flavor of WOF.

At high temperatures, Welsh onion produces a unique flavor in the frying oil. A high temperature promotes certain chemical reactions, including the caramelization of carbohydrates and the Maillard reaction between carbonyls and amines, as well as accelerated lipid degradation and oxidation, leading to changes in the aroma and texture of fried foods [4]. Also, various interactions between precursors and their degradation products in Welsh onion and oil can occur during heating. Moreover, hazardous compounds can also be generated during the heating process. Polycyclic aromatic hydrocarbons (PAHs) and acrylamide are known as typical hazardous compounds that can be formed when frying vegetables that contain carbohydrates and proteins.

PAHs constitute a large class of organic compounds that are produced through the incomplete combustion or pyrolysis of organic matter [5]. PAHs are known to act as endocrine disruptors and carcinogens, and 16 types of PAHs have been designated as major harmful substances by the US Environmental Protection Agency (EPA). In the food processing industry, various heating processes, including frying, grilling, smoking, drying, baking, and ohmic-infrared cooking, contribute to the formation of PAHs [6]. In rats, acrylamide was found to have a potency comparable to PAHs formed in fried or grilled meats [7]. 

Acrylamide is mainly formed by the Maillard reaction between asparagine and reducing sugars in raw materials when heated to temperatures higher than 120 °C [8]. Asparagine and sugars can be released from Welsh onion into the oil during frying. Also, oil vigorously produces various reactive carbonyl compounds via peroxides, which readily results in the formation of acrylamide [9]. However, hazardous compounds generated during the heating process when using various oils to fry *Allium* species have not been reported previously. Studies of hazardous compounds produced when frying Welsh onion are urgently needed to ensure the safety of the associated food industry.

During a high heat treatment process, it is expected that the generation of some hazardous substances, as well as flavor components occurs, which can affect the quality and safety of Welsh onion fried. The purpose of this study was to investigate the effects of different temperatures and types of vegetable oils on the flavor profiles of WOF and to evaluate hazardous compounds, such as PAHs and acrylamide, produced when heating to a high temperature. It is well known that heating temperatures can critically affect the formations of both volatile compounds and some hazardous substances. Corn, canola, soybean, and palm oils, which have different compositions of fatty acids, were applied as heating oils to compare the differences in the volatile compound profiles and the formation of hazardous substances. 

## 2. Materials and Methods

### 2.1. Materials and Chemicals

Fresh Welsh onion (*Allium fistulosum* L.), soybean oil, corn oil, canola oil, and palm oil were purchased from a local market in Seoul, Korea. Welsh onions were cultivated in Jindo-gun, Jeollanam-do, Korea. (E)-Dec-2-enal was purchased from Fluka (Buchs, Switzerland). The internal standard (1S,2R,4S)-1,7,7-trimethylbicyclo [2.2.1]heptan-2-ol ((-)-borneol) and the authentic standard compounds were obtained from Sigma Aldrich (St. Louis, MO, USA). All solvents were HPLC-grade (J.T.Baker, Phillipsburg, NJ, USA).

Eight polycyclic aromatic hydrocarbons (PAHs) standards, such as benzo[a]anthracene (B[a]A), chrysene (CHR), benzo[b]fluoranthene (B[b]F), benzo[k]fluoranthene (B[k]F), benzo[a]pyrene (B[a]P), indeno[1,2,3-c,d]pyrene (I[c,d]P), dibenz[a,h]anthracene (D[a,h]A), and benzo[g,h,i]perylene (B[g,h,i]P), and 2 internal standard compounds, such as benzo[a]pyrene-d12 (B[a]P-d12) and chrysene-d12 (CHR-d12), were purchased from Sigma-Aldrich (St. Louis, MO, USA). Acrylamide (>99%) and 13C3-acrylamide (>99%) were supplied from Cambridge Isotope Laboratories (Andover, MA, USA).

Sep-pak florisil cartridge^®^ (1 g, 6 mL), Strata-X (200 mg, 6 mL), and Bond Elut AccuCAT (200 mg, 3 mL) solid-phase extraction (SPE) cartridges were purchased from Waters (Milford, MA, USA), Phenomenex (Torrance, CA, USA), and Agilent Technologies (Santa Clara, CA, USA), respectively. Polytetrafluoroethylene (PTEF) membrane filters (13.0 mm, 0.45 μm pore size) and Polyvinylidene fluoride (PVDF) syringe filters were obtained from Whatman^TM^ (Clifton, NJ, USA) and Futecs Co. (Daejeon, Korea), respectively.

### 2.2. Preparation of WOF Samples

The stalks of Welsh onions were cut into approximately 1 cm pieces. The Welsh onion (50.0 g) and vegetable oil (75.0 g, soybean, corn, canola, and palm each oil) were mixed and transferred to a 250 mL stainless steel cylinder (Ilshinautoclave, Daejeon, Korea). The cylinders were heated at different temperatures (140, 165, and 190 °C) in a drying oven (Eyela NDO-600SD, Rikakikai Co. Ltd., Tokyo, Japan) for 90 min. Zhang et al. [3] demonstrated that frying at temperatures in the range of 140 °C to 165 °C were the most significant for flavor formation of Welsh onion. We also studied the heating temperature of 190 °C, which represents a typical frying condition. The heating time was chosen in the preliminary study, which showed a significant volatiles formation at those temperatures. The reaction was immediately stopped by cooling in cold water. The samples were divided into residue and oil by paper filtration. Then oil was obtained and transferred into a 50 mL amber laboratory bottle (Simax, New York, NY, USA), which was sealed tightly after nitrogen flushing and stored at −80 °C until analysis. Each of the samples was prepared in triplicate.

### 2.3. Analysis of Volatile Compounds

Solid-phase micro-extraction (SPME) was applied to extract volatile compounds of WOF. Five grams of the WOF sample was weighed in a 20 mL headspace vial. The internal standard [5 μL, (-)-borneol, 500 mg/L in methanol] was added into a sealed vial. The samples were maintained at 40 °C for 30 min to reach the equilibrium state and adsorbed for 30 min using SPME fiber coated with polydimethylsiloxane/divinylbenzene fiber (PDMS/DVB, 65 μm, Supelco, Bellefonte, PA, USA).

The analysis of volatile compounds was carried out using a 7890A gas chromatograph (GC, Agilent Technologies, Santa Clara, CA, USA) with a 5977B mass selective detector (MS, Agilent Technologies). Separations were performed on a fused silica capillary column (DB-5ms, 30 m length × 0.25 mm i.d. × 0.25 µm film thickness, J&W Scientific, Folsom, CA, USA). Helium (99.999%) was used as the carrier gas at a constant flow rate of 0.8 mL/min. The initial GC oven temperature was maintained at 40 °C for 5 min, raised to 80 °C at 1.5 °C/min and to 130 °C at 3 °C/min, and finally, increased to 200 °C at 10 °C/min and held for 10 min. The temperatures of the injector and transfer line were 230 °C and 260 °C, respectively, and the split-less injection mode was applied. The mass spectrometer was operated in the electron impact (EI) ion source mode at 70 eV and a scanning range of 35–350 atomic mass units (a.m.u.).

Identification of volatile compounds was positively confirmed by comparing retention times and mass spectral data with those of authentic standard compounds. When the authentic compounds were not available, each volatile compound was identified on the base of their mass spectral using NIST.08 and Wiley.9. The retention indices (RIs) of volatile compounds were calculated with n-alkane from C_7_ to C_30_ as an external standard. The quantification of each peak was performed by comparing its peak area with that of an internal standard compound on GC-MS total ion chromatograms. The experiment was conducted in triplicate.

### 2.4. Analysis of PAHs

The contents of PAHs were determined using a method established in the total diet study of The Korean Ministry of Food and Drug Safety (KMFDS) with minor modifications (KMFDS, 2019). Ten grams of the WOF samples were weighed in a 250 mL glass bottle. As internal standard compounds (ISTD), a mixture of CHR-d12 and B[a]P-d12 were prepared at 10.0 µg/mL (*w/v*) in methylene chloride and 10 µL of the internal standards mixture was spiked into the sample. The analysis of PAHs was carried out using a 7890B GC with a 5977A MS (Agilent Technologies). Separations were performed on the fused silica capillary column of DB-5ms (30 m length × 0.25 mm i.d. × 0.25 µm film thickness, J&W Scientific). Helium was used as the carrier gas at a constant flow rate of 1mL/min. The initial GC oven temperature was maintained at 80 °C for 1 min, raised to 245 °C at 6 °C/min and increased to 270 °C at 30 °C/min and held for 15 min. The post-run temperature was kept at 270 °C for 10 min. The injector and transfer line were heated at 300 °C and 250 °C, respectively, and the injection volume was 1.0 µL with splitless injection mode. The EI mode was applied at 70 eV. The ions selected for the qualification and quantitation of 8 PAHs and 2 ISTDs were as follows: B[a]A (*m*/*z* 228 and *m*/*z* 226, 229), CHR (*m*/*z* 240 and *m*/*z* 236, 241), B[b]F (*m*/*z* 252 and *m*/*z* 250, 253), B[b]K (*m*/*z* 252 and *m*/*z* 250, 253), B[a]P (*m*/*z* 252 and *m*/*z* 250, 253), I[c,d]P (*m*/*z* 276 and *m*/*z* 277, 274), D[a,h]A (*m*/*z* 278 and *m*/*z* 279, 276), B[g,h,i]P (*m*/*z* 276 and *m*/*z* 277, 274), CHR-d12 (*m*/*z* 240 and *m*/*z* 236, 241), and B[a]P-d12 (*m*/*z* 264 and *m*/*z* 263, 265). The amounts of PAHs were determined by comparing their peak areas to those of the ISTDs, CHR-d12, and B[a]P-d12.

### 2.5. Analysis of Acrylamide

The analytical method employed for acrylamide determination was based on the KMFDS method with minor modifications (KMFDS, 2019). The oil sample (1 g) was weighed into a 50 mL conical tube, and double-distilled water (9 mL) and acrylamide-d3 (ISTD, 1 mL, 200 ng/mL) were added to the tube. The extraction was conducted by shaking the tube at 250 rpm for 20 min. Then the sample was centrifuged at 26,912× *g* for 5 min. The supernatant was filtered through a 0.45 µm PVDF syringe filter. The sample purification was performed with two SPE columns in a series. An SPE column (Strata-X) was conditioned with methanol (3.5 mL) followed by water (3.5 mL). An aliquot of the sample (1.5 mL) was loaded onto the SPE cartridge, followed by water (0.5 mL). The eluent was discarded. Then, an additional 1.5 mL water was passed through the column, and the eluent was collected. Another SPE column (Bond Elut AccuCAT) was conditioned with 2.5 mL methanol and 2.5 mL water in a series. An aliquot (0.5 mL) of the eluate collected from the Strata-X SPE column was loaded on the preconditioned Bond Elut AccuCAT SPE column. Then, an additional 1 mL of the eluate from the Strata-X column was introduced onto the Bond Elut AccuCAT SPE column. The eluent was collected in a vial. High-performance liquid chromatography-tandem mass spectrometry (HPLC-MS/MS) was carried out using a Shimadzu 30A HPLC system coupled to a Shimadzu MS8040 MS/MS system (Shimadzu, Kyoto, Japan) in a positive electrospray ionization mode (5000 V). The mobile phase used was a solution consisting of 0.1% acetic acid in 0.5% methanol in water (MS grade). The multiple reaction monitoring (MRM) transitions for the quantitation and qualification of acrylamide were *m*/*z* 72 > 55 and *m*/*z* 72 > 27, respectively. The multiple reaction monitoring (MRM) transitions for the quantitation and qualification of acrylamide-d3 (ISTD) were *m*/*z* 75 > 58 and *m*/*z* 75 > 29, respectively. A C18 column (Kinetex polar C18 column, 150 mm × 2.1 mm i.d., 2.6 µm particle size, Phenomenex) was used for the separation of acrylamide. The quantitation was based on a standard calibration curve obtained from peak areas vs. concentrations of the authentic acrylamide.

### 2.6. Statistical Analysis

The experimental results were expressed as means ± standard deviation. Analysis of variance (ANOVA) was conducted using IMB SPSS program (IBM Corp., Armonk, NY, USA) to evaluate statistically significant differences in volatile compounds, PAHs, and acrylamide of WOF at different heating temperatures. Duncan’s multiple range test was carried out on the level of significant different level (*p* < 0.05).

## 3. Results and Discussion

### 3.1. Volatile Changes of WOF According to Types of Vegetable Oil and Frying Temperatures

SPME coupled with GC-MS was applied to compare the differences in volatile compounds produced according to different temperatures and types of vegetable oils. Table S1 lists the volatile compounds identified from WOF samples and their relative peak areas and RIs. There were 162 volatile compounds identified across all WOF samples: 5 acids, 14 alcohols, 28 aldehydes, 1 benzene derivative, 3 esters, 19 furans/furanones, 20 hydrocarbons, 20 ketones, 5 lactones, 6 nitrogen-containing compounds, 7 pyrazines, 3 phenols, and 31 sulfur-containing compounds. In general, the total content of volatile compounds in all WOF samples increased with the temperature, with the increase being the smallest for soybean oil.

**Figure 1:**
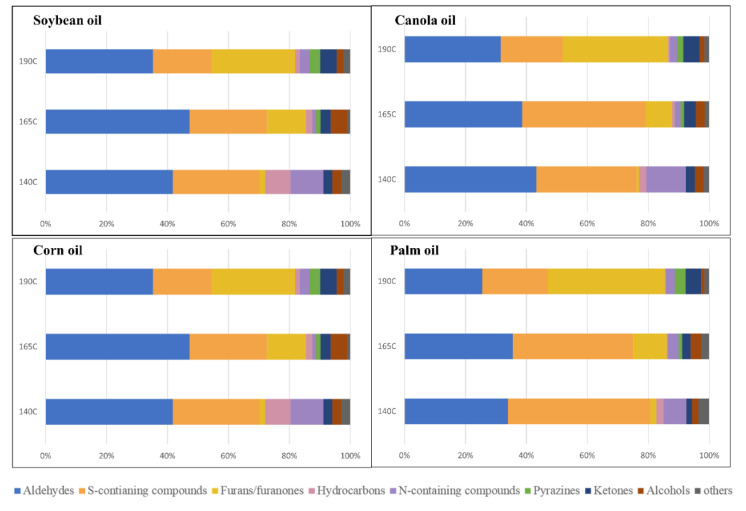
Quantitative composition of identified volatile compounds in WOF (Welsh onion fried) samples according to chemical functional groups. Each stacked bar is 100% wide. Each figure represents the changes in volatile compounds of WOF in different vegetable oils according to the heating temperatures.

Figure 1 shows the quantitative composition of identified volatile compounds in the WOF samples according to chemical functional groups. Chemical groups, such as aldehydes, furans/furanones, and sulfur-containing compounds, were major volatile groups in all WOF samples, which is consistent with a previous study [3]. They demonstrated that these compounds were the most important contributors to the flavor of WOF.

The composition of each chemical group showed different changes depending on the temperatures; for examples, the proportions of aldehydes, sulfur-containing compounds, and hydrocarbons decreased, while those of furans/furanones and ketones increased with increasing temperature. These changes in chemical groups could contribute to the flavor characteristics of WOF. Aldehydes and sulfur-containing compounds are generally composed of low odor thresholds and unique odor descriptions. In particular, furans/furanones, which have cooked and caramel flavor notes, were found to contribute significantly to the characteristic flavor formation of deep-fried oil with Welsh onion [3].

**Figure 2:**
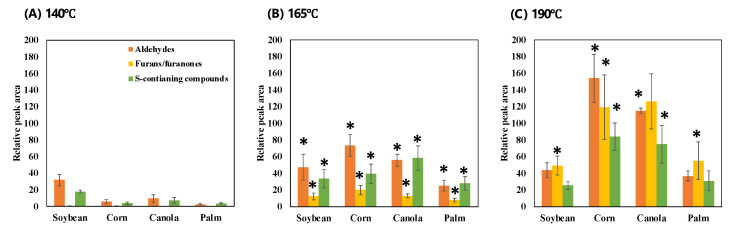
Changes of major chemical groups (aldehydes, furans/furanones, and sulfur-containing compounds) in WOF (Welsh onion fried) samples depending on different temperatures: (**A**) WOF prepared at 140 °C, (**B**) at 165 °C, (**C**) at 190 °C. Asterisk indicates a statistically significant difference (*p*-value < 0.05) in the changes of chemical groups compared to the samples prepared at a lower temperature (140 or 165 °C).

Figure 2 shows the changes in major chemical groups, such as aldehydes, furans/furanones, and sulfur-containing compounds, according to the types and temperatures of the frying oil. Aldehydes can be the dominant odor compounds in oils used to fry Allium species, mainly due to their low odor thresholds [1] and unique odor descriptions, such as waxy, green, and grassy. Both aliphatic and branched-chain aldehydes are generally formed via specific reactions, such as lipid oxidation or Strecker degradation. Lipid oxidation begins with the generation of hydroperoxide and progresses via mechanisms involving free radicals. There are high contents of oleic acids and linoleic acids in corn and canola oils, which are easily oxidized to hydroperoxides [10]. Hydroperoxides are then decomposed into aldehydes, acids, hydrocarbons, and other compounds [2]. This study found that the contents of aldehydes were the highest at 190 °C in WOF based on corn, canola, and palm oils, but not for soybean oil. High temperatures can enhance lipid oxidation by increasing the breakdown of hydroperoxides, which produces free radicals [11]. This increases the oxidation rate of fatty acids, which enhances aldehyde formation. The total amount of aldehydes was lowest in WOF prepared using palm oil. The proportion of saturated fatty acids relative to unsaturated fatty acids is higher in palm oil than in other vegetable oils, including soybean, corn, and canola oils. Compared with saturated fatty acids, unsaturated fatty acids, such as linoleic acid and linolenic acid, are more susceptible to oxidation due to the presence of multiple double bonds [12].

Aldehydes, such as hexanal, 2,4-decadienal, and (E)-oct-2-enal, are lipid-degraded compounds of linoleic acid [13], and their contents were largest in corn oil rich in linoleic acid. Octanal, nonanal, (E)-dec-2-enal, and (E)-undec-2-enal were found in considerable amounts in canola oil with a high oleic acid content. Cao et al. [14] reported that octanal, nonanal, 2-decenal, and 2-undecenal were the characteristic carbonyls for oleic- acid-rich oils via the thermal oxidation of oleic acid triglycerides. In the present study, the contents of certain aldehydes, such as hexanal, (2E,4E)-deca-2,4-dinal, 3-methylbutanal, and 2-phenylacetaldehyde, increased significantly in all WOF samples with increasing temperature. Hexanal was generated via thermal oxidative degradation of unsaturated fatty acids. 3-Methylbutanal was formed via the thermally induced Strecker degradation of leucine [15], whereas 2-phenylacetaldehyde was derived from phenylalanine via a similar pathway [16].

The results of this study indicated that the contents of furans/furanones increased with temperature. Furans/furanones are produced via various pathways, such as Maillard reaction, carbohydrate pyrolysis, and lipid oxidation, that are significantly influenced by temperature. The contents of furan-2-carbaldehyde (furfural), furan-2-ylmethanol (furfuryl alcohol), 1-(furan-2-yl)ethenone (2-acetylfuran), 5-methylfuran-2-carbaldehyde (5-methylfurfural), and 5-(hydroxyl-methyl)furan-2-carbaldehyde (HMF), 2-pentylfuran, and 1-(furan-2-yl)-2-hydroxy-ethanone increased when WOF was exposed to a higher temperature. In particular, furfural and HMF, which are mainly produced by the degradation of monosaccharides, were major furan derivatives. In addition, they can originate from carbonyl compounds produced by the oxidation of lipids [17].

Among vegetable oils, the contents of furans/furanones were higher in WOF prepared using corn and canola oils, which have high proportions of unsaturated fatty acids. Furans can be generated from various precursors, including unsaturated fatty acids [18]. Shen et al. [19] demonstrated the content of furans related to heating time in the linoleic model system. Unsaturated fatty acids are more prone to oxidation than are saturated fatty acids, and hydroperoxides and their oxides decompose faster to produce more carbonyl compounds. The abundance of furan derivatives in WOF prepared using corn and canola oils might be explained by the large amounts of unsaturated fatty acids in these oils.

The characteristic aromas of Allium species are commonly attributed to sulfur-containing volatiles [20]. We found that the main sulfur-containing compounds of the WOF samples included (methyldisulfanyl)methane (dimethyl disulfide), 1-(methyl-disulfanyl)propane (methyl propyl disulfide), 1-(propyldisulfanyl)propane (dipropyl disulfide), (methyltrisulfanyl)methane (dimethyl trisulfide), and (methyl-tetrasulfanyl)methane (dimethyl tetrasulfide). These compounds can be generated by the thermal degradation of sulfur-containing amino acids [3]. The volatile chemicals in the species of Allium genus are produced through the hydrolysis of nonvolatile sulfur-containing compounds, termed S-alk(en)yl-L-cysteine sulfoxides (CSs) [19]. The main CSs in Welsh onion are S-propenyl-L-cysteine sulfoxide, S-methyl-L-cysteine sulfoxide, and S-propyl-L-cysteine sulfoxide [21]. CSs are stored in the cytosol of storage mesophyll cells, and during thermal or enzymatic tissue damage, they produce their corresponding sulfenic acids, which are further converted into various sulfur-containing compounds, including dimethyl sulfides, methyl propyl sulfides, and dipropyl sulfides [22]. In addition, many thiophenes and thiols are produced from thermal interactions between fatty aldehydes and hydrogen sulfide produced by sulfur amino acid degradation [23]. Sulfur-containing compounds in WOF samples were the most abundant at 190 °C, except when using soybean oil. Disulfides and polysulfides constituted the main sulfur-containing compounds. The contents of thiols and thiophenes in WOF produced using corn and canola oils were higher than those produced using soybean and palm oils. This might be explained by corn and canola oils containing larger amounts of unsaturated fatty acids, which can readily lead to the production of fatty aldehydes.

The contents of pyrazines were also sensitively changed by heat treatment. It is known that nitrogen-containing compounds are primarily formed from nitrogenous substances being degraded from amino acids, peptides, and proteins with carbohydrate-degraded compounds [24]. The present results indicated that the amount of alkyl pyrazines, including 2-methylpyrazine, 2,6-dimethylpyrazine, and 2-ethylpyrazine, increased with temperature. Pyrazines are considered to be mainly responsible for roasted flavors [25], and so the increased contents of pyrazines might enhance such flavors in WOF.

Phenols, such as 2-methoxyphenol (guaiacol) and 4-ethyl-2-methoxyphenol, (4-ethylguaiacol), were only generated in WOF samples at 190 °C. These volatile compounds can be formed in two ways: thermal decarboxylation and enzymatic carboxylase activity [26]. A previous study [27] demonstrated that the formation of volatile phenols was positively correlated with the storage period and the heating temperature. Phenols generally contribute to “smoky”, “woody”, and “spicy” odor notes and also have extremely low threshold values, such as 1.8 (for 2-methoxyphenol) and 37 ug/kg (for 4-ethyl-2-methoxyphenol) in oil, respectively [28,29]. Therefore, the formation of volatile phenols, which have unique odor notes as well as low threshold values, may be responsible for the characteristic flavors of WOF samples.

### 3.2. Hazardous Compounds in WOF

The formation of potential carcinogens needs to be examined in fried foods because the frying process involves exposure to high temperatures. PAHs and acrylamide are known as typical carcinogenic compounds in fried foods. This study investigated how the changes in PAHs and acrylamide contents in WOF samples varied with the different frying temperatures and types of frying oil.

PAHs are a class of organic compounds that are produced through the incomplete combustion or pyrolysis of organic matter [5]. Monounsaturated hydrocarbons in oils and fats undergo aromatization and dehydrocyclization, favoring the formation of PAHs and, therefore, also the contamination of foodstuffs processed using these oils [6,30]. Table 1 lists the amounts of eight PAHs in WOF samples for different frying temperatures and for different types of vegetable oils. All PAHs were detected in WOF produced using soybean oil, while no PAHs were found in WOF samples prepared using corn and canola oils. Moreover, some PAHs, such as B[b]F, I[c,d]P, D[a,h]A, and B[g,h,i]P, were found in this study in WOF samples produced using palm oil. In general, unsaturated fatty acids are considered to produce more PAHs due to their structure, making it easier to form free radicals and/or small molecules compared with saturated fatty acids [31]. However, in this study, PAHs were not detected in WOF samples produced using corn and canola oils, which have higher proportions of unsaturated fatty acids than soybean and palm oils. In general, all PAHs studied were found to be not detected or at trace (or very low) levels regardless of oil types and heating temperatures. The type of vegetable oil was sensitive to the formation of some PAHs, although it was not significantly affected by heating temperatures in this study.

Acrylamide in the WOF samples was also quantitatively determined using LC-MS/MS (Table 2). Certain reactive carbonyls, such as fatty acid oxidation products and sugar degradation products, can react with asparagine when heating Welsh onion, resulting in the formation of acrylamide [32]. In this study, acrylamide was not detected in all WOF samples at 140 °C. However, it was formed only in soybean oil at 165 °C, whereas it was found in all samples at 190 °C. Based on these results, it might be considered that the formation of acrylamide was considerably influenced by both heating temperatures and oil types.

To ensure the safety of PAHs in edible oils, the European Union (EU) has established a limit of 10 µg/kg for the total amount of PAH4 (B[a]A, CHR, B[b]F, and B[a]P) and 2 µg/kg for B[a]P in vegetable oils. The KMFDS has similarly established a limit of 2 µg/kg for B[a]P in all edible oils. The amounts of PAHs in all of the samples analyzed in this study were within the EU and KMFDS limits. Acrylamide formation in foodstuffs varies greatly depending on the temperature and duration of processing. International organizations such as the World Health Organization (WHO) and the Codex Alimentarius Commission (CODEX) have not established international standards for acrylamide in edible oils. In 2021 the KMFDS set recommended standards for acrylamide in infant food (≤0.3 mg/kg), cereals (≤0.3 mg/kg), snacks (≤1 mg/kg), French fries (≤1 mg/kg), coffee beans (≤0.8 mg/kg), processed grains (≤1 mg/kg), and instant foodstuffs (≤1 mg/kg), but these do not address edible oils. Under the conditions used in the present study, acrylamide was detected at 36.46 µg/kg in the WOF samples. The present results can therefore be used to develop legal regulations on hazardous compounds for frying oils used with Allium species, as well as forming the basis for research into the industrial processing of Welsh onion fried.

## 4. Conclusions

This study investigated the effects of the temperature (140, 165, and 190 °C) and type of vegetable frying oil (soybean, corn, canola, and palm oils) on the formation of volatile compounds and hazardous compounds, such as PAHs and acrylamide. In general, the degree of temperature affected the generation of acrylamide as well as that of volatile compounds, whereas the formations of volatile compounds and both hazardous compounds, PAHs and acrylamide, were highly related to the type of vegetable oils. Frying heating process, using various oils, including soybean, corn, canola, and palm oil, have been applied in industry as well as home cooking. Thus, it is important to understand the impacts of temperatures and type of oils on the generations of volatile compounds and hazardous substances.

Volatile compounds of WOF samples were formed via a variety of chemical reactions, such as thermal oxidative degradation of lipids and the Maillard reaction. Certain chemical groups, such as aldehydes, furans/furanones, and sulfur-containing compounds were identified as the major compounds in WOF samples. The contents of volatile compounds, in particular, aldehydes derived from fatty acids, and thiol and thiophenes, were highly dependent on the composition of unsaturated fatty acids in the frying oils.

When the contents of PAHs and acrylamide produced in Welsh onion fried using vegetable oils at different temperatures were determined, that of acrylamide showed a significant difference depending on both heating temperatures and type of vegetable oils, while those of PAHs were affected only by the type of vegetable oils. In general, PAHs were detected at lower than the EU maximum tolerable limit, and acrylamide was detected below 36.46 µg/kg. These results can be used as basic data for the industrial process of frying Welsh onion and utilized to develop legal regulations on the production of acrylamide when frying Welsh onion products.

## Figures and Tables

**Table 1:** The formation of polycyclic aromatic hydrocarbons (PAHs) in WOF (Welsh onion fried) samples at 190 °C.

PAHs	Heating Temp.	Concentrations ^1^ (μg/kg)			
		Soybean Oil	Corn Oil	Canola Oil	Palm Oil
B[a]A	140 °C	0.65 ± 0.02 b ^2^	N.D. a	N.D. a	N.D. a
	165 °C	N.D. ^3^ a	N.D. a	N.D. a	N.D. a
	190 °C	0.71 ± 0.01b	N.D. a	N.D. a	N.D. a
CHR	140 °C	0.64 ± 0.02 b	N.D. a	N.D. a	N.D. a
	165 °C	N.D. a	N.D. a	N.D. a	N.D. a
	190 °C	0.61 ± 0.04 b	N.D. a	N.D. a	N.D. a
B[b]F	140 °C	1.26 ± 0.17 b	N.D. a	N.D. a	Trace ^4^ a
	165 °C	N.D. a	N.D. a	N.D. a	0.28 ± 0.01 b
	190 °C	1.39 ± 0.03 b	N.D. a	N.D. a	0.26 ± 0.00 b
B[k]F	140 °C	0.72 ± 0.05 b	N.D. a	N.D. a	N.D. a
	165 °C	N.D. a	N.D. a	N.D. a	N.D. a
	190 °C	0.75 ± 0.02 b	N.D. a	N.D. a	N.D. a
B[a]P	140 °C	1.38 ± 0.05 b	N.D. a	N.D. a	trace a
	165 °C	0.59 ± 0.04 a	N.D. a	N.D. a	trace a
	190 °C	1.42 ± 0.05 b	N.D. a	N.D. a	trace a
I[c,d]P	140 °C	0.41 ± 0.05 b	N.D. a	N.D. a	0.36 ± 0.03 b
	165 °C	trace a	N.D. a	N.D. a	0.30 ± 0.03 ab
	190 °C	0.51 ± 0.04 c	N.D. a	N.D. a	0.32 ± 0.01 a
D[a,h]A	140 °C	0.15 ± 0.01 a	N.D. a	N.D. a	0.16 ± 0.01 a
	165 °C	0.17 ± 0.01 ab	N.D. a	N.D. a	0.19 ± 0.05 a
	190 °C	0.19 ± 0.02 b	N.D. a	N.D. a	0.19 ± 0.01 a
B[g,h,i]P	140 °C	0.48 ± 0.06 b	N.D. a	N.D. a	0.36 ± 0.02 a
	165 °C	0.11 ± 0.00 a	N.D. a	N.D. a	0.34 ± 0.05 a
	190 °C	0.53 ± 0.03 b	N.D. a	N.D. a	0.37 ± 0.05 a

^1^ All results are expressed as mean ± standard deviation for three replicates. ^2^ Mean values with different letters (a and b) show significant differences (*p* < 0.05) within a row using Duncan’s multiple comparison test. ^3^ Not detected, ^4^ trace < LOQ.

**Table 2:** The formation of acrylamide in WOF (Welsh onion fried) samples at 190 °C.

Compound	Heating Temp.	Concentrations ^1^ (μg/kg)			
		Soy Bean Oil	Corn Oil	Canola Oil	Palm Oil
Acrylamide	140 °C	N.D. ^3^ a ^2^	N.D. a	N.D. a	N.D. a
	165 °C	7.31 ± 0.80 b	N.D. a	N.D. a	N.D. a
	190 °C	14.72 ± 2.68 c	21.74 ± 8.98 b	25.38 ± 3.76 b	36.46 ± 12.83 b

^1^ All results are expressed as mean ± standard deviation for three replicates. ^2^ Mean values with different letters show significant differences (*p* < 0.05) within a row using Duncan’s multiple comparison test. ^3^ Not detected.

## Data Availability

Data is contained within the article or supplementary material.

## Supplementary Materials

The following supporting information can be downloaded at: https://www.mdpi.com/article/10.3390/foods11091335/s1, Table S1: Volatile compounds identified in welsh onion (*Allium fistulosum* L.) fried
