# Supplementary material for: Study on Volatile Profiles, Polycyclic Aromatic Hydrocarbons, and Acrylamide Formed in Welsh Onion (Allium fistulosum L.) Fried in Vegetable Oils at Different Temperatures
Source: Foods. 2022 May 4;11(9):1335. doi: 10.3390/foods11091335 (PMC9100245; doi:10.3390/foods11091335)
Supplement: Supplementary file 1 [file foods-11-01335-s001.zip › foods-1664086-supplementary.pdf]

**Supplementary Material**

# Evaluation for the quality and safety of welsh onion (*Allium fistulosum* L.) fried depending on temperature and type of oils

**Hye-Min Kim [1], Min-Kyung Park [1], Soo-Jeong Mun [1], Mun-Yhung Jung [2],**

**Sang-Mi Lee [3] and Young-Suk Kim [1,*]**

**Table S1**. Volatile compounds identified in welsh onion (*Allium fistulosum* L.) fried

| RI[1)] | Volatile compounds | Heating temp. | Relative peak area[2)] | | | | ID[3)] |
|---|---|---|---|---|---|---|---|
| | | | Frying oil | | | | |
| | | | Soybean oil | Corn oil | Canola oil | Palm oil | |
| ***Acids*** | | | | | | | |
| 605 | acetic acid | 140°C | N.D.[4)] a[5)] | 0.051±0.044 a | 0.025±0.043 a | 0.023±0.022 a | A |
| | | 165°C | 0.364±0.195 b | 0.184±0.085 b | N.D. a | N.D. a | |
| | | 190°C | N.D. a | N.D. a | N.D. a | N.D. a | |
| 1,183 | octanoic acid | 140°C | N.D. a | N.D. a | N.D. a | N.D. a | A |
| | | 165°C | N.D. a | N.D. a | N.D. a | N.D. a | |
| | | 190°C | N.D. a | N.D. a | 0.321±0.075 b | N.D. a | |
| 1,275 | nonanoic acid | 140°C | 0.363±0.241 a | N.D. a | N.D. a | N.D. a | A |
| | | 165°C | 0.087±0.091 a | N.D. a | N.D. a | 0.429±0.234 b | |
| | | 190°C | 0.145±0.034 a | 0.769±0.293 b | 0.287±0.189 b | 0.123±0.078 a | |
| 1,564 | dodecanoic acid | 140°C | 0.154±0.071 b | 0.034±0.025 b | N.D. a | N.D. a | A |
| | | 165°C | N.D. a | N.D. a | 0.285±0.280 a | 0.090±0.047 a | |
| | | 190°C | N.D. a | N.D. a | 0.054±0.047 a | 0.053±0.065 a | |
| 1,956 | hexadecanoic acid | 140°C | N.D. a | N.D. a | N.D. a | N.D. a | B |
| | | 165°C | 0.125±0.031 b | N.D. a | N.D. a | N.D. a | |
| | | 190°C | N.D. a | N.D. a | N.D. a | N.D. a | |
| ***Alcohols*** | | | | | | | |
| 679 | pent-1-en-3-ol | 140°C | 0.274±0.079 b | N.D. a | 0.196±0.101 a | N.D. a | A |
| | | 165°C | N.D. a | N.D. a | 0.708±0.111 b | N.D. a | |
| | | 190°C | N.D. a | 0.515±0.146 b | 0.963±0.113 c | 0.137±0.024 b | |
| 761 | pentan-1-ol | 140°C | N.D. a | 0.040±0.015 a | N.D. a | N.D. a | A |
| | | 165°C | 1.757±0.648 b | 2.494±0.349 b | 0.794±0.085 b | 0.623±0.298 b | |
| | | 190°C | 0.454±0.067 a | 3.133±0.659 b | 1.628±0.122 c | 0.449±0.107 b | |
| 784 | 3-methylbutan-1-ol | 140°C | 0.150±0.022 b | N.D. a | N.D. a | N.D. a | A |
| | | 165°C | N.D. a | N.D. a | N.D. a | N.D. a | |
| | | 190°C | N.D. a | N.D. a | N.D. a | N.D. a | |

**Table S1.** (Continued)

| RI[1)] | Volatile compounds | Heating temp. | Relative peak area[2)] | | | | ID[3)] |
|---|---|---|---|---|---|---|---|
| | | | Frying oil | | | | |
| | | | Soybean oil | Corn oil | Canola oil | Palm oil | |
| ***Alcohols*** | | | | | | | |
| | | 140°C | 0.145±0.025 b | N.D. a | N.D. a | N.D. a | |
| 902 | 2-butoxyethanol | 165°C | N.D. a | N.D. a | N.D. a | N.D. a | A |
| | | 190°C | N.D. a | N.D. a | N.D. a | N.D. a | |
| | | 140°C | N.D. a | N.D. a | N.D. a | N.D. a | |
| 969 | heptan-1-ol | 165°C | 0.623±0.524 a | 0.974±0.147 b | 0.649±0.070 b | 0.190±0.079 b | A |
| | | 190°C | 0.222±0.110 a | 0.886±0.272 b | 1.580±0.219 c | 0.295±0.077 b | |
| | | 140°C | 0.510±0.099 a | 0.140±0.053 a | 0.120±0.048 a | 0.021±0.020 a | |
| 979 | oct-1-en-3-ol | 165°C | 1.022±0.358 b | 1.548±0.163 b | 0.411±0.101 b | 0.150±0.048 b | A |
| | | 190°C | 0.386±0.045 a | 1.765±0.540 b | 0.693±0.157 c | 0.119±0.010 b | |
| | | 140°C | 0.231±0.119 a | 0.138±0.048 a | 0.218±0.096 a | 0.144±0.075 a | |
| 997 | 2-(2-ethoxyethoxy)ethanol | 165°C | 0.467±0.328 a | 0.400±0.079 a | 0.743±0.630 a | 0.955±0.631 b | A |
| | | 190°C | 0.661±0.776 a | 1.805±1.431 a | 0.664±0.180 a | 0.189±0.056 a | |
| | | 140°C | N.D. a | N.D. a | N.D. a | N.D. a | |
| 1,028 | 2-ethylhexan-1-ol | 165°C | N.D. a | N.D. a | N.D. a | 0.237±0.084 b | A |
| | | 190°C | N.D. a | N.D. a | N.D. a | N.D. a | |
| | | 140°C | 0.224±0.209 a | N.D. a | N.D. a | N.D. a | |
| 1,070 | octan-1-ol | 165°C | 0.789±0.395 b | 1.121±0.230 b | 0.586±0.269 b | 0.132±0.019 b | A |
| | | 190°C | N.D. a | N.D. a | N.D. a | N.D. a | |
| | | 140°C | 0.871±0.208 c | 0.071±0.017 a | 0.131±0.041 b | N.D. a | |
| 1,091 | 2,5-dimethylcyclohexan-1-ol | 165°C | 0.555±0.177 b | 1.321±0.215 c | 0.230±0.076 b | N.D. a | B |
| | | 190°C | N.D. a | 0.512±0.140 b | N.D. a | 0.240±0.056 b | |
| | | 140°C | N.D. a | N.D. a | N.D. a | N.D. a | |
| 1,183 | 2-(2-butoxyethoxy)ethanol | 165°C | N.D. a | N.D. a | N.D. a | 0.139±0.069 b | A |
| | | 190°C | N.D. a | 0.304±0.267 a | N.D. a | N.D. a | |

**Table S1.** (Continued)

| RI[1)] | Volatile compounds | Heating temp. | Relative peak area[2)] | | | | ID[3)] |
|---|---|---|---|---|---|---|---|
| | | | Frying oil | | | | |
| | | | Soybean oil | Corn oil | Canola oil | Palm oil | |
| ***Alcohols*** | | | | | | | |
| | | 140°C | N.D. a | N.D. a | N.D. a | N.D. a | |
| 1,293 | 3,7-dimethylocta-2,6-dien-1-ol | 165°C | N.D. a | N.D. a | N.D. a | N.D. a | B |
| | | 190°C | N.D. a | N.D. a | 0.138±0.086 b | N.D. a | |
| | | 140°C | N.D. a | 0.034±0.022 b | N.D. a | N.D. a | |
| 1,464 | 2-methyloxolan-2-ol | 165°C | N.D. a | N.D. a | N.D. a | N.D. a | C |
| | | 190°C | N.D. a | N.D. a | N.D. a | N.D. a | |
| ***Aldehydes*** | | | | | | | |
| | | 140°C | 2.180±0.818 b | 0.591±0.200 a | 1.072±0.517 a | 0.431±0.272 a | |
| <600 | propanal | 165°C | 2.151±0.776 b | 3.638±0.492 b | 2.916±0.509 b | 2.067±1.557 a | A |
| | | 190°C | 0.268±0.147 a | 4.783±1.006 b | 5.049±0.430 c | 1.979±0.546 a | |
| | | 140°C | N.D. a | N.D. a | N.D. a | N.D. a | |
| <600 | 2-methylpropanal | 165°C | 0.289±0.174 a | 0.292±0.113 a | 0.366±0.025 a | 0.237±0.063 a | A |
| | | 190°C | 2.514±2.477 a | 3.271±1.147 b | 3.291±0.705 b | 1.866±0.713 b | |
| | | 140°C | N.D. a | N.D. a | 0.048±0.027 a | N.D. a | |
| 641 | (E)-but-2-enal | 165°C | 0.148±0.032 a | 0.326±0.129 b | 0.269±0.042 b | N.D. a | B |
| | | 190°C | 0.567±0.542 a | 0.273±0.090 b | 0.590±0.055 c | 0.130±0.030 b | |
| | | 140°C | N.D. a | N.D. a | N.D. a | N.D. a | |
| 646 | 3-methylbutanal | 165°C | 1.068±0.543 b | 2.217±0.931 a | 3.030±1.110 b | 2.002±0.816 b | A |
| | | 190°C | 3.567±0.412 c | 12.986±1.684 b | 9.639±1.350 c | 5.289±0.799 c | |
| | | 140°C | N.D. a | 0.027±0.012 a | N.D. a | N.D. a | |
| 656 | 2-methylbutanal | 165°C | N.D. a | N.D. a | 0.652±0.341 a | 0.429±0.053 a | A |
| | | 190°C | 1.984±0.414 b | 5.112±0.545 b | 3.885±0.788 b | 2.107±0.486 b | |
| | | 140°C | 1.352±0.138 a | 0.269±0.128 a | 0.339±0.163 a | 0.084±0.021 a | |
| 698 | pentanal | 165°C | 2.356±0.794 b | 3.199±0.687 b | 1.860±0.210 a | 1.139±0.478 b | A |
| | | 190°C | 1.557±0.137 ab | 6.401±1.440 c | 5.150±1.751 b | 1.861±0.675 b | |

**Table S1.** (Continued)

| RI[1)] | Volatile compounds | Heating temp. | Relative peak area[2)] | | | | ID[3)] |
|---|---|---|---|---|---|---|---|
| | | | Frying oil | | | | |
| | | | Soybean oil | Corn oil | Canola oil | Palm oil | |
| ***Aldehydes*** | | | | | | | |
| 732 | (E)-2-methylbut-2-enal | 140°C | 0.250±0.106 b | 0.032±0.017 b | 0.080±0.084 a | 0.068±0.024 b | B |
| | | 165°C | N.D. a | N.D. a | N.D. a | N.D. a | |
| | | 190°C | N.D. a | N.D. a | N.D. a | N.D. a | |
| 745 | (E)-pent-2-enal | 140°C | 0.161±0.019 b | 0.024±0.008 a | 0.160±0.073 a | N.D. a | A |
| | | 165°C | 0.316±0.091 c | 0.204±0.091 b | 0.836±0.075 b | 0.140±0.020 b | |
| | | 190°C | N.D. a | 0.379±0.113 c | 1.061±0.104 c | N.D. a | |
| 800 | hexanal | 140°C | 9.098±1.760 b | 1.953±0.661 a | 1.968±0.962 a | 0.391±0.228 a | A |
| | | 165°C | 8.983±2.412 b | 18.849±2.854 b | 8.479±2.418 b | 4.752±1.954 b | |
| | | 190°C | 4.402±0.257 a | 26.613±5.606 c | 12.641±0.766 c | 2.953±0.163 b | |
| 824 | (E)-2-methylpent-2-enal | 140°C | 2.369±0.470 b | 0.701±0.190 b | 1.454±0.593 b | 0.901±0.248 a | A |
| | | 165°C | N.D. a | N.D. a | 1.794±0.462 b | 2.135±0.824 b | |
| | | 190°C | N.D. a | N.D. a | N.D. a | N.D. a | |
| 846 | (E)-hex-2-enal | 140°C | 0.150±0.012 b | 0.042±0.019 a | 0.062±0.030 a | N.D. a | A |
| | | 165°C | N.D. a | N.D. a | 0.379±0.027 b | 0.154±0.061 b | |
| | | 190°C | 0.267±0.086 c | 0.920±0.188 b | 0.587±0.071 c | 0.094±0.017 b | |
| 889 | 2-ethyl-3-methylbutanal | 140°C | N.D. a | N.D. a | N.D. a | N.D. a | C |
| | | 165°C | N.D. a | N.D. a | N.D. a | N.D. a | |
| | | 190°C | N.D. a | N.D. a | 0.237±0.035 b | 0.061±0.009 b | |
| 901 | heptanal | 140°C | 0.573±0.056 a | 0.145±0.079 a | 0.353±0.221 a | 0.081±0.022 a | A |
| | | 165°C | 2.130±1.780 a | 2.963±0.348 b | 3.300±0.791 b | 1.615±0.686 b | |
| | | 190°C | 0.551±0.090 a | 3.758±0.538 c | 4.495±0.231 c | 1.192±0.236 b | |
| 952 | (E)-hept-2-enal | 140°C | 2.666±0.161 a | 0.983±0.551 a | 0.766±0.376 a | 0.103±0.031 a | B |
| | | 165°C | 10.329±3.313 b | 14.714±2.887 b | 3.546±0.855 b | 1.503±0.355 c | |
| | | 190°C | 3.971±0.387 a | 27.343±5.326 c | 5.230±0.693 c | 0.788±0.125 b | |

**Table S1.** (Continued)

| RI[1)] | Volatile compounds | Heating temp. | Relative peak area[2)] | | | | ID[3)] |
|---|---|---|---|---|---|---|---|
| | | | Frying oil | | | | |
| | | | Soybean oil | Corn oil | Canola oil | Palm oil | |
| ***Aldehydes*** | | | | | | | |
| | | 140°C | 1.515±0.771 a | 0.182±0.083 a | 0.535±0.373 a | 0.139±0.014 a | |
| 1,001 | octanal | 165°C | 3.294±2.744 a | 4.433±0.955 b | 4.761±1.005 b | 2.128±0.578 b | A |
| | | 190°C | 1.114±0.221 a | 5.426±1.657 b | 9.244±1.050 c | 2.759±0.330 b | |
| | | 140°C | 0.666±0.290 a | N.D. a | 1.299±0.674 a | N.D. a | |
| 1,007 | (2E,4E)-hepta-2,4-dienal | 165°C | 3.719±0.814 c | 1.983±0.490 b | 7.360±1.237 b | 0.166±0.018 b | B |
| | | 190°C | 2.555±0.433 b | 1.462±0.399 b | 11.882±0.868 c | 0.258±0.079 b | |
| | | 140°C | 0.186±0.015 a | 0.109±0.108 a | 0.072±0.050 a | 0.084±0.020 a | |
| 1,036 | 2-phenylacetaldehyde | 165°C | 3.223±1.351 a | 5.237±2.227 a | 4.182±1.358 b | 2.671±0.732 a | B |
| | | 190°C | 10.633±3.254 b | 23.579±6.277 b | 15.555±2.231 c | 8.841±2.329 b | |
| | | 140°C | 1.145±0.088 a | 0.247±0.060 a | 0.208±0.174 a | 0.031±0.007 a | |
| 1,054 | (E)-oct-2-enal | 165°C | 2.977±1.070 b | 5.417±0.687 b | 1.140±0.140 b | 0.495±0.071 c | A |
| | | 190°C | 0.872±0.145 a | 4.427±1.359 b | 2.180±0.457 c | 0.366±0.067 b | |
| | | 140°C | 9.106±3.023 b | 0.308±0.068 a | 0.997±0.649 a | 0.361±0.209 a | |
| 1,101 | nonanal | 165°C | 3.563±1.894 a | 4.816±1.150 b | 5.724±1.197 b | 2.510±0.313 b | A |
| | | 190°C | 2.211±0.348 a | 8.461±2.345 c | 12.888±1.693 c | 3.393±0.262 c | |
| | | 140°C | 0.092±0.081 a | 0.036±0.013 a | N.D. a | N.D. a | |
| 1,160 | (E)-non-2-enal | 165°C | 0.293±0.215 ab | 0.622±0.243 a | 0.410±0.020 b | 0.166±0.010 b | A |
| | | 190°C | 0.452±0.076 b | 1.535±0.458 b | 1.329±0.107 c | 0.381±0.086 c | |
| | | 140°C | 0.156±0.110 b | 0.048±0.043 a | N.D. a | N.D. a | |
| 1,202 | decanal | 165°C | N.D. a | N.D. b | 0.201±0.045 b | 0.118±0.016 b | A |
| | | 190°C | 0.152±0.029 b | 0.402±0.123 b | 0.416±0.097 c | 0.248±0.032 c | |
| | | 140°C | N.D. a | N.D. a | N.D. a | N.D. a | |
| 1,210 | (2E,4E)-nona-2,4-dienal | 165°C | 0.082±0.078 a | 0.205±0.026 a | N.D. a | N.D. a | A |
| | | 190°C | N.D. a | 0.349±0.147 b | N.D. a | 0.062±0.005 b | |

**Table S1.** (Continued)

| RI[1)] | Volatile compounds | Heating temp. | Relative peak area[2)] Frying oil Soybean oil | Corn oil | Canola oil | Palm oil | ID[3)] |
|---|---|---|---|---|---|---|---|
| ***Aldehydes*** | | | | | | | |
| | | 140°C | 0.214±0.118 a | 0.049±0.025 a | 0.190±0.124 a | N.D. a | |
| 1,258 | (E)-dec-2-enal | 165°C | 0.658±0.538 a | 1.187±0.225 a | 1.821±0.208 b | 0.326±0.063 b | A |
| | | 190°C | 0.621±0.167 a | 3.435±1.110 b | 3.982±0.271 c | 0.605±0.060 c | |
| | | 140°C | N.D. a | 0.072±0.063 a | 0.081±0.039 a | N.D. a | |
| 1,311 | (2E,4E)-deca-2,4-dienal | 165°C | 1.169±0.466 a | 2.981±0.651 a | 2.085±0.367 b | 0.313±0.073 b | A |
| | | 190°C | 5.342±1.222 b | 12.169±3.984 b | 4.657±0.863 c | 1.142±0.253 c | |
| | | 140°C | N.D. a | N.D. a | 0.061±0.013 a | N.D. a | |
| 1,359 | (E)-undec-2-enal | 165°C | N.D. a | N.D. a | 0.581±0.147 b | 0.140±0.010 b | A |
| | | 190°C | 0.167±0.039 b | 0.686±0.272 b | 1.205±0.109 c | 0.235±0.041 c | |
| | | 140°C | 0.187±0.247 a | 0.035±0.026 a | N.D. a | N.D. a | |
| 1,404 | dodecanal | 165°C | N.D. a | N.D. a | 0.097±0.019 b | N.D. a | A |
| | | 190°C | N.D. a | 0.191±0.102 b | N.D. a | 0.027±0.024 a | |
| | | 140°C | N.D. a | 0.049±0.035 b | N.D. a | N.D. a | |
| 1,815 | octadecanal | 165°C | N.D. a | N.D. a | 0.104±0.007 b | N.D. a | C |
| | | 190°C | N.D. a | N.D. a | N.D. a | N.D. a | |
| ***Benzene derivatives*** | | | | | | | |
| | | 140°C | 0.643±0.166 b | 0.122±0.044 a | 0.243±0.090 a | 0.163±0.041 a | |
| 1,395 | 1-methoxy-4-[(Z)-prop-1-enyl]benzene | 165°C | N.D. a | N.D. a | 0.559±0.201 b | 0.482±0.057 b | C |
| | | 190°C | 0.414±0.146 b | 0.575±0.163 b | 0.210±0.035 a | 0.118±0.031 a | |
| ***Esters*** | | | | | | | |
| | | 140°C | N.D. a | N.D. a | N.D. a | N.D. a | |
| 863 | 2-oxopropyl acetate | 165°C | N.D. a | N.D. a | N.D. a | N.D. a | B |
| | | 190°C | N.D. a | 0.705±0.217 b | 0.727±0.226 b | 0.401±0.177 b | |

**Table S1.** (Continued)

| RI[1)] | Volatile compounds | Heating temp. | Relative peak area[2)] | | | | ID[3)] |
|---|---|---|---|---|---|---|---|
| | | | Frying oil | | | | |
| | | | Soybean oil | Corn oil | Canola oil | Palm oil | |
| ***Esters*** | | | | | | | |
| 863 | 2-oxopropyl acetate | 140°C | N.D. a | N.D. a | N.D. a | N.D. a | B |
| | | 165°C | N.D. a | N.D. a | N.D. a | N.D. a | |
| | | 190°C | N.D. a | 0.705±0.217 b | 0.727±0.226 b | 0.401±0.177 b | |
| 1,338 | (1-hydroxy-2,4,4-trimethylpentan-3-yl) 2-methylpropanoate | 140°C | N.D. a | 0.072±0.044 a | 0.047±0.050 a | 0.039±0.033 a | C |
| | | 165°C | 0.088±0.152 a | 0.166±0.169 a | 0.207±0.085 a | 0.222±0.110 b | |
| | | 190°C | 0.176±0.190 a | 0.442±0.341 a | 0.104±0.090 a | N.D. a | |
| 1,364 | (3-hydroxy-2,4,4-trimethylpentyl) 2-methylpropanoate | 140°C | N.D. a | 0.102±0.063 a | 0.094±0.063 a | 0.058±0.064 a | C |
| | | 165°C | 0.172±0.231 a | 0.154±0.116 a | 0.450±0.246 b | 0.411±0.251 b | |
| | | 190°C | 0.198±0.343 a | 0.777±0.687 a | 0.118±0.102 a | 0.044±0.015 a | |
| ***Furans and furanones*** | | | | | | | |
| 601 | 2-methylfuran | 140°C | N.D. a | N.D. a | N.D. a | N.D. a | A |
| | | 165°C | N.D. a | N.D. a | N.D. a | N.D. a | |
| | | 190°C | N.D. a | 0.480±0.433 a | 1.148±0.672 b | 0.348±0.210 b | |
| 700 | 2-ethylfuran | 140°C | N.D. a | N.D. a | N.D. a | N.D. a | B |
| | | 165°C | 0.161±0.055 b | 0.238±0.017 b | 1.444±0.326 b | N.D. a | |
| | | 190°C | N.D. a | N.D. a | N.D. a | N.D. a | |
| 742 | 3-methylfuran | 140°C | N.D. a | N.D. a | N.D. a | N.D. a | C |
| | | 165°C | N.D. a | N.D. a | N.D. a | N.D. a | |
| | | 190°C | 0.194±0.051 b | 1.329±0.441 b | 1.529±0.898 b | 0.937±0.400 b | |
| 780 | 5-methyl-2,3-dihydrofuran | 140°C | N.D. a | N.D. a | N.D. a | N.D. a | C |
| | | 165°C | N.D. a | N.D. a | N.D. a | N.D. a | |
| | | 190°C | N.D. a | N.D. b | N.D. a | 0.045±0.010 b | |

**Table S1.** (Continued)

| RI[1)] | Volatile compounds | Heating temp. | Relative peak area[2)] | | | | ID[3)] |
|---|---|---|---|---|---|---|---|
| | | | Frying oil | | | | |
| | | | Soybean oil | Corn oil | Canola oil | Palm oil | |
| ***Furans and furanones*** | | | | | | | |
| 825 | furan-2-carbaldehyde (furfural) | 140°C | N.D. a | N.D. a | N.D. a | N.D. a | A |
| | | 165°C | 5.505±2.201 b | 8.442±2.079 a | 5.963±1.397 a | 4.460±1.472 a | |
| | | 190°C | 20.461±2.165 c | 53.552±16.416 b | 70.018±14.422 b | 31.551±13.558 b | |
| 851 | furan-2-ylmethanol (furfuryl alcohol) | 140°C | N.D. a | 0.095±0.123 a | 0.076±0.034 a | 0.051±0.011 a | A |
| | | 165°C | 3.668±1.484 b | 5.433±1.659 b | 2.929±0.509 b | 1.871±0.618 b | |
| | | 190°C | 3.006±0.659 b | 9.781±2.778 c | 5.605±0.392 c | 3.217±0.863 c | |
| 859 | 5-methyl-3H-furan-2-one | 140°C | N.D. a | N.D. a | N.D. a | N.D. a | A |
| | | 165°C | N.D. a | N.D. a | N.D. a | N.D. a | |
| | | 190°C | N.D. a | 0.246±0.070 b | 0.330±0.097 b | 0.148±0.063 b | |
| 905 | 1-(furan-2-yl)ethanone | 140°C | N.D. a | N.D. a | N.D. a | N.D. a | A |
| | | 165°C | N.D. a | 0.997±0.454 a | 0.644±0.277 a | 0.435±0.049 a | |
| | | 190°C | 2.613±0.291 b | 7.990±2.943 b | 7.884±2.491 b | 3.895±1.679 b | |
| 928 | 2-methyl-2H-furan-5-one | 140°C | N.D. a | N.D. a | N.D. a | N.D. a | B |
| | | 165°C | N.D. a | N.D. a | N.D. a | N.D. a | |
| | | 190°C | N.D. a | N.D. a | 0.286±0.037 b | 0.162±0.071 b | |
| 954 | 5-methylfuran-2-carbaldehyde | 140°C | N.D. a | N.D. a | N.D. a | 0.056±0.018 a | A |
| | | 165°C | 0.496±0.104 a | 0.618±0.212 a | 0.433±0.112 a | 0.345±0.069 a | |
| | | 190°C | 4.459±0.820 b | 10.445±4.523 b | 14.694±6.385 b | 6.978±3.995 b | |
| 975 | 2,4-dihydroxy-2,5-dimethylfuran-3-one | 140°C | N.D. a | N.D. a | N.D. a | N.D. a | B |
| | | 165°C | N.D. a | N.D. a | N.D. a | N.D. a | |
| | | 190°C | N.D. a | 0.351±0.080 b | N.D. a | 0.123±0.030 b | |
| 986 | 2-pentylfuran | 140°C | 0.701±0.051 a | 0.145±0.091 a | 0.104±0.050 a | 0.045±0.009 a | A |
| | | 165°C | 2.148±0.572 b | 3.472±0.725 b | 1.217±0.088 b | 0.635±0.156 b | |
| | | 190°C | 1.756±0.095 b | 7.090±1.853 c | 2.756±0.408 c | 0.789±0.151 b | |

**Table S1.** (Continued)

| RI[1)] | Volatile compounds | Heating temp. | Relative peak area[2)] | | | | ID[3)] |
|---|---|---|---|---|---|---|---|
| | | | Frying oil | | | | |
| | | | Soybean oil | Corn oil | Canola oil | Palm oil | |
| ***Furans and furanones*** | | | | | | | |
| 1,025 | 3,4-dimethylfuran-2,5-dione | 140°C | N.D. a | N.D. a | N.D. a | N.D. a | A |
| | | 165°C | N.D. a | N.D. a | N.D. a | N.D. a | |
| | | 190°C | 0.283±0.074 b | 0.513±0.173 b | 0.381±0.017 b | 0.204±0.110 b | |
| 1,027 | 1-(5-methylfuran-2-yl)ethanone | 140°C | N.D. a | N.D. a | N.D. a | N.D. a | A |
| | | 165°C | N.D. a | N.D. a | N.D. a | N.D. a | |
| | | 190°C | 0.424±0.132 b | 0.799±0.304 b | 0.329±0.116 b | 0.142±0.092 b | |
| 1,030 | 2-(furan-2-yl)furan | 140°C | N.D. a | N.D. a | N.D. a | N.D. a | B |
| | | 165°C | N.D. a | N.D. a | N.D. a | N.D. a | |
| | | 190°C | N.D. a | N.D. a | 0.126±0.036 b | 0.046±0.012 b | |
| 1,033 | furan-2,5-dicarbaldehyde | 140°C | N.D. a | N.D. a | N.D. a | N.D. a | B |
| | | 165°C | N.D. a | N.D. a | N.D. a | N.D. a | |
| | | 190°C | 1.073±0.201 b | 2.573±1.240 b | 2.322±0.515 b | 1.082±0.482 b | |
| 1,057 | 4-hydroxy-2,5-dimethylfuran-3-one (furaneol) | 140°C | N.D. a | N.D. a | N.D. a | N.D. a | B |
| | | 165°C | N.D. a | N.D. a | N.D. a | N.D. a | |
| | | 190°C | 0.523±0.133 b | 0.577±0.645 a | 0.568±0.346 b | 0.145±0.084 b | |
| 1,072 | 1-(furan-2-yl)-2-hydroxyethanone | 140°C | N.D. a | N.D. a | N.D. a | N.D. a | B |
| | | 165°C | N.D. a | N.D. a | N.D. a | N.D. a | |
| | | 190°C | 3.093±0.759 b | 8.336±3.594 b | 7.106±2.061 b | 2.610±0.567 b | |
| 1,225 | 5-(hydroxymethyl)furan-2-carbaldehyde (5-hydroxymethylfurfural) | 140°C | N.D. a | N.D. a | N.D. a | N.D. a | B |
| | | 165°C | 0.265±0.149 a | 0.761±0.417 a | 0.187±0.066 a | 0.081±0.073 a | |
| | | 190°C | 11.511±7.675 b | 15.220±8.468 b | 11.250±6.596 b | 2.785±1.418 b | |
| 1,296 | 2-benzofuran-1,3-dione | 140°C | N.D. a | 0.023±0.010 b | N.D. a | N.D. a | B |
| | | 165°C | N.D. a | N.D. a | N.D. a | N.D. a | |
| | | 190°C | N.D. a | N.D. a | N.D. a | N.D. a | |

**Table S1.** (Continued)

| RI[1)] | Volatile compounds | Heating temp. | Relative peak area[2)] | | | | ID[3)] |
|---|---|---|---|---|---|---|---|
| | | | Frying oil | | | | |
| | | | Soybean oil | Corn oil | Canola oil | Palm oil | |
| ***Hydrocarbons*** | | | | | | | |
| 646 | (3E)-2-methylpenta-1,3-diene | 140°C | 0.364±0.065 b | 0.156±0.103 b | 0.210±0.095 b | 0.083±0.034 b | B |
| | | 165°C | N.D. a | N.D. a | N.D. a | N.D. a | |
| | | 190°C | N.D. a | N.D. a | N.D. a | N.D. a | |
| 687 | 1,2-dimethylcyclopentane | 140°C | N.D. a | N.D. a | N.D. a | N.D. a | C |
| | | 165°C | N.D. a | N.D. a | 0.132±0.053 b | N.D. a | |
| | | 190°C | N.D. a | 0.212±0.075 b | 0.139±0.019 b | N.D. a | |
| 787 | oct-1-ene | 140°C | N.D. a | 0.025±0.017 a | N.D. a | N.D. a | B |
| | | 165°C | N.D. a | N.D. a | 0.151±0.046 b | N.D. a | |
| | | 190°C | N.D. a | 0.662±0.169 b | 0.171±0.010 b | N.D. a | |
| 805 | (E)-oct-2-ene | 140°C | N.D. a | 0.083±0.104 a | N.D. a | N.D. a | B |
| | | 165°C | 0.308±0.066 b | 0.393±0.152 b | N.D. a | N.D. a | |
| | | 190°C | N.D. a | N.D. a | N.D. a | N.D. a | |
| 811 | (Z)-oct-2-ene | 140°C | N.D. a | 0.029±0.032 a | N.D. a | N.D. a | B |
| | | 165°C | 0.170±0.033 b | 0.198±0.016 a | 0.137±0.062 ab | N.D. a | |
| | | 190°C | 0.227±0.022 c | 1.293±0.386 b | 0.250±0.149 b | N.D. a | |
| 821 | (3E)-octa-1,3-diene | 140°C | N.D. a | N.D. a | N.D. a | N.D. a | B |
| | | 165°C | N.D. a | N.D. a | N.D. a | N.D. a | |
| | | 190°C | N.D. a | 0.565±0.290 b | N.D. a | N.D. a | |
| 954 | 2,5,6-trimethyldecane | 140°C | 0.615±0.136 b | 0.150±0.070 b | N.D. a | N.D. a | C |
| | | 165°C | N.D. a | N.D. a | N.D. a | N.D. a | |
| | | 190°C | N.D. a | N.D. a | N.D. a | N.D. a | |
| 967 | 2,2,6-trimethyloctane | 140°C | 0.204±0.034 b | 0.033±0.014 b | N.D. a | N.D. a | B |
| | | 165°C | N.D. a | N.D. a | N.D. a | N.D. a | |
| | | 190°C | N.D. a | N.D. a | N.D. a | N.D. a | |

**Table S1.** (Continued)

| RI[1)] | Volatile compounds | Heating temp. | Relative peak area[2)] | | | | ID[3)] |
|---|---|---|---|---|---|---|---|
| | | | Frying oil | | | | |
| | | | Soybean oil | Corn oil | Canola oil | Palm oil | |
| ***Hydrocarbons*** | | | | | | | |
| | | 140°C | 0.713±0.102 c | 0.116±0.036 b | N.D. a | N.D. a | |
| 1,019 | 2,2,8-trimethyldecane | 165°C | 0.295±0.081 b | 0.490±0.082 c | N.D. a | N.D. a | C |
| | | 190°C | N.D. a | N.D. a | N.D. a | N.D. a | |
| | | 140°C | N.D. a | N.D. a | N.D. a | N.D. a | |
| 1,023 | (3E)-3-ethyl-2-methylhexa-1,3-diene | 165°C | 0.463±0.158 c | 0.626±0.131 b | 0.188±0.056 b | 0.112±0.028 b | B |
| | | 190°C | 0.203±0.075 b | 0.553±0.191 b | 0.168±0.025 b | 0.088±0.033 b | |
| | | 140°C | 0.299±0.070 b | 0.045±0.011 b | N.D. a | N.D. a | |
| 1,025 | 2,2,4,6,6-pentamethylheptane | 165°C | N.D. a | N.D. a | N.D. a | N.D. a | B |
| | | 190°C | N.D. a | N.D. a | N.D. a | N.D. a | |
| | | 140°C | 0.982±0.175 c | 0.177±0.072 b | 0.144±0.053 a | 0.076±0.047 a | |
| 1,027 | 3,7-dimethylnonane | 165°C | 0.292±0.087 b | 0.450±0.113 c | 0.207±0.065 b | 0.100±0.087 a | B |
| | | 190°C | N.D. a | N.D. a | N.D. b | N.D. a | |
| | | 140°C | 0.295±0.116 b | 0.064±0.006 a | N.D. a | N.D. a | |
| 1,043 | 1-ethyl-2-methylcyclopentane | 165°C | N.D. a | N.D. a | N.D. a | N.D. a | B |
| | | 190°C | N.D. a | 1.442±0.922 b | 0.442±0.135 b | 0.211±0.083 b | |
| | | 140°C | 0.782±0.117 c | 0.127±0.043 b | N.D. a | N.D. a | |
| 1,046 | 2,6-dimethyloctane | 165°C | 0.240±0.069 b | 0.412±0.070 c | N.D. a | N.D. a | C |
| | | 190°C | N.D. a | N.D. a | N.D. a | N.D. a | |
| | | 140°C | 0.282±0.048 c | 0.042±0.012 a | N.D. a | N.D. a | |
| 1,048 | 2-methyldecane | 165°C | 0.119±0.033 b | 0.209±0.036 b | N.D. a | N.D. a | B |
| | | 190°C | N.D. a | N.D. a | N.D. a | N.D. a | |
| | | 140°C | 0.620±0.076 b | 0.088±0.025 a | N.D. a | N.D. a | |
| 1,059 | 2,6,8-trimethyldecane | 165°C | 0.260±0.076 a | 0.397±0.060 b | N.D. a | N.D. a | B |
| | | 190°C | 0.261±0.040 a | 0.596±0.210 b | N.D. a | N.D. a | |

**Table S1.** (Continued)

| RI[1)] | Volatile compounds | Heating temp. | Relative peak area[2)] | | | | ID[3)] |
|---|---|---|---|---|---|---|---|
| | | | Frying oil | | | | |
| | | | Soybean oil | Corn oil | Canola oil | Palm oil | |
| ***Hydrocarbons*** | | | | | | | |
| | | 140°C | 0.342±0.029 b | 0.058±0.017 a | N.D. a | N.D. a | |
| 1,069 | 3,3,6-trimethyldecane | 165°C | N.D. a | N.D. a | N.D. a | N.D. a | C |
| | | 190°C | N.D. a | 0.375±0.124 b | N.D. a | N.D. a | |
| | | 140°C | N.D. a | N.D. a | 0.118±0.067 a | N.D. a | |
| 1,075 | 3-methylbicyclo[4.1.0]heptane | 165°C | N.D. a | N.D. a | 0.361±0.081 b | N.D. a | C |
| | | 190°C | N.D. a | N.D. a | 0.320±0.128 b | N.D. a | |
| | | 140°C | N.D. a | N.D. a | N.D. a | N.D. a | |
| 1,094 | 3,3-dimethylhex-1-ene | 165°C | N.D. a | N.D. a | N.D. a | N.D. a | C |
| | | 190°C | 0.235±0.069 b | 0.942±0.421 b | 0.244±0.068 b | 0.113±0.030 b | |
| | | 140°C | N.D. a | N.D. a | 0.049±0.020 a | 0.028±0.009 b | |
| 1,197 | dodecane | 165°C | N.D. a | N.D. ab | N.D. a | N.D. a | A |
| | | 190°C | N.D. a | 0.176±0.064 b | 0.154±0.041 b | N.D. a | |
| | | 140°C | N.D. a | N.D. a | N.D. a | N.D. a | |
| 1,433 | 1-ethyl-1,4-dimethylcyclohexane | 165°C | N.D. a | N.D. a | N.D. a | N.D. a | C |
| | | 190°C | N.D. a | 0.223±0.204 a | N.D. a | N.D. a | |
| ***Ketones*** | | | | | | | |
| | | 140°C | 0.317±0.082 a | N.D. a | N.D. a | N.D. a | |
| <600 | butane-2,3-dione | 165°C | 0.499±0.142 a | 0.593±0.213 a | 0.598±0.044 a | 0.534±0.107 a | A |
| | | 190°C | 0.613±0.662 a | 2.966±1.419 b | 4.176±1.198 b | 1.636±0.621 b | |
| | | 140°C | N.D. a | N.D. a | N.D. a | N.D. a | |
| 661 | 1-hydroxypropan-2-one | 165°C | 0.220±0.109 a | 0.426±0.159 a | 0.192±0.170 a | 0.395±0.158 a | A |
| | | 190°C | 2.895±0.213 b | 5.258±1.098 b | 3.999±0.971 b | 1.893±0.601 b | |
| | | 140°C | 0.415±0.091 c | 0.095±0.043 a | 0.234±0.108 a | 0.126±0.040 a | |
| 709 | 3-hydroxybutan-2-one | 165°C | 0.217±0.077 b | 0.247±0.056 a | 0.451±0.097 b | 0.532±0.156 b | B |
| | | 190°C | N.D. a | 1.375±0.343 b | 1.188±0.117 c | 0.455±0.165 b | |

**Table S1.** (Continued)

| RI[1)] | Volatile compounds | Heating temp. | Relative peak area[2)] | | | | ID[3)] |
|---|---|---|---|---|---|---|---|
| | | | Frying oil | | | | |
| | | | Soybean oil | Corn oil | Canola oil | Palm oil | |
| ***Ketones*** | | | | | | | |
| <600 | butane-2,3-dione | 140°C | 0.317±0.082 a | N.D. a | N.D. a | N.D. a | A |
| | | 165°C | 0.499±0.142 a | 0.593±0.213 a | 0.598±0.044 a | 0.534±0.107 a | |
| | | 190°C | 0.613±0.662 a | 2.966±1.419 b | 4.176±1.198 b | 1.636±0.621 b | |
| 661 | 1-hydroxypropan-2-one | 140°C | N.D. a | N.D. a | N.D. a | N.D. a | A |
| | | 165°C | 0.220±0.109 a | 0.426±0.159 a | 0.192±0.170 a | 0.395±0.158 a | |
| | | 190°C | 2.895±0.213 b | 5.258±1.098 b | 3.999±0.971 b | 1.893±0.601 b | |
| 709 | 3-hydroxybutan-2-one | 140°C | 0.415±0.091 c | 0.095±0.043 a | 0.234±0.108 a | 0.126±0.040 a | B |
| | | 165°C | 0.217±0.077 b | 0.247±0.056 a | 0.451±0.097 b | 0.532±0.156 b | |
| | | 190°C | N.D. a | 1.375±0.343 b | 1.188±0.117 c | 0.455±0.165 b | |
| 783 | 3-methylpentan-2-one | 140°C | N.D. a | N.D. a | N.D. a | N.D. a | A |
| | | 165°C | 0.129±0.053 b | 0.242±0.043 a | 0.220±0.013 b | N.D. a | |
| | | 190°C | N.D. a | 0.636±0.212 b | 0.304±0.044 c | 0.126±0.030 b | |
| 874 | cyclopent-4-ene-1,3-dione | 140°C | N.D. a | N.D. a | N.D. a | N.D. a | B |
| | | 165°C | N.D. a | 0.301±0.055 a | 0.124±0.037 a | N.D. a | |
| | | 190°C | 0.648±0.063 b | 1.921±0.618 b | 1.761±0.470 b | 0.946±0.366 b | |
| 886 | heptan-2-one | 140°C | 0.178±0.035 a | 0.051±0.025 a | N.D. a | 0.021±0.019 a | A |
| | | 165°C | 0.502±0.324 a | 1.128±0.312 b | 0.355±0.089 b | 0.185±0.062 b | |
| | | 190°C | 0.233±0.064 a | 1.523±0.275 b | 0.590±0.095 c | 0.180±0.029 b | |
| 920 | cyclopentane-1,2-dione | 140°C | N.D. a | N.D. a | N.D. a | N.D. a | C |
| | | 165°C | N.D. a | N.D. a | N.D. a | N.D. a | |
| | | 190°C | N.D. a | 0.363±0.133 b | 0.426±0.062 b | 0.208±0.055 b | |
| 941 | 1-(cyclohexen-1-yl)ethanone | 140°C | 0.191±0.019 a | 0.040±0.023 a | 0.179±0.091 a | N.D. a | B |
| | | 165°C | 0.541±0.146 b | 0.442±0.100 a | 0.689±0.244 b | N.D. a | |
| | | 190°C | 0.310±0.060 a | 1.533±0.361 b | 0.654±0.028 b | 0.085±0.030 b | |

**Table S1.** (Continued)

| RI[1)] | Volatile compounds | Heating temp. | Relative peak area[2)] | | | | ID[3)] |
|---|---|---|---|---|---|---|---|
| | | | Frying oil | | | | |
| | | | Soybean oil | Corn oil | Canola oil | Palm oil | |
| ***Ketones*** | | | | | | | |
| 944 | 1-cyclohex-3-en-1-ylethanone | 140°C | 0.086±0.074 a | N.D. a | 0.172±0.092 a | N.D. a | C |
| | | 165°C | 0.254±0.089 b | N.D. a | 0.757±0.276 b | N.D. a | |
| | | 190°C | 0.235±0.043 b | N.D. a | 0.616±0.055 b | N.D. a | |
| 973 | oct-1-en-3-one | 140°C | 0.181±0.024 a | 0.076±0.019 a | 0.062±0.025 a | N.D. a | A |
| | | 165°C | 0.586±0.290 b | 0.722±0.130 b | 0.179±0.012 ab | N.D. a | |
| | | 190°C | 0.340±0.098 a | 0.812±0.381 b | 0.284±0.108 b | N.D. a | |
| 984 | 2-methyloctan-3-one | 140°C | 0.637±0.022 a | 0.033±0.011 a | N.D. a | N.D. a | B |
| | | 165°C | 3.408±0.656 b | 0.332±0.036 b | 0.133±0.016 b | N.D. a | |
| | | 190°C | 4.789±0.482 c | 0.478±0.161 b | 0.233±0.083 c | N.D. a | |
| 1,019 | 2-hydroxy-3-methylcyclopent-2-en-1-one | 140°C | N.D. a | N.D. a | N.D. a | N.D. a | B |
| | | 165°C | N.D. a | N.D. a | N.D. a | N.D. a | |
| | | 190°C | 0.560±0.079 b | 1.512±0.668 b | 1.010±0.388 b | 0.423±0.191 b | |
| 1,031 | cyclopentane-1,3-dione | 140°C | N.D. a | N.D. a | N.D. a | N.D. a | C |
| | | 165°C | N.D. a | N.D. a | N.D. a | N.D. a | |
| | | 190°C | N.D. a | N.D. a | 0.250±0.138 b | N.D. a | |
| 1,055 | dodecane-6,7-dione | 140°C | 0.431±0.076 b | 0.074±0.016 b | N.D. a | N.D. a | C |
| | | 165°C | N.D. a | N.D. a | N.D. a | N.D. a | |
| | | 190°C | N.D. a | N.D. a | N.D. a | N.D. a | |
| 1,080 | 3-ethyl-2-hydroxycyclopent-2-en-1-one | 140°C | N.D. a | N.D. a | N.D. a | N.D. a | A |
| | | 165°C | N.D. a | N.D. a | N.D. a | N.D. a | |
| | | 190°C | N.D. a | 0.329±0.092 b | 0.358±0.123 b | 0.141±0.074 b | |
| 1,087 | nonan-2-one | 140°C | N.D. a | N.D. a | N.D. a | N.D. a | A |
| | | 165°C | N.D. a | N.D. a | N.D. a | N.D. a | |
| | | 190°C | N.D. a | N.D. a | N.D. a | 0.076±0.021 b | |

**Table S1.** (Continued)

| RI[1)] | Volatile compounds | Heating temp. | Relative peak area[2)] | | | | ID[3)] |
|---|---|---|---|---|---|---|---|
| | | | Frying oil | | | | |
| | | | Soybean oil | Corn oil | Canola oil | Palm oil | |
| ***Ketones*** | | | | | | | |
| 1,140 | 3,5-dihydroxy-6-methyl-2,3-dihydropyran-4-one | 140°C | N.D. a | N.D. a | N.D. a | N.D. a | B |
| | | 165°C | 0.321±0.158 a | 0.631±0.108 a | 0.162±0.172 a | 0.155±0.049 a | |
| | | 190°C | 2.298±0.665 b | 4.716±3.375 b | 2.568±1.279 b | 0.693±0.464 b | |
| 1,189 | decan-2-one | 140°C | N.D. a | N.D. a | N.D. a | N.D. a | A |
| | | 165°C | N.D. a | N.D. a | N.D. a | N.D. a | |
| | | 190°C | N.D. a | N.D. a | 0.250±0.019 b | 0.070±0.009 b | |
| 1,381 | bicyclo[2.2.2]oct-5-en-2-one | 140°C | 0.187±0.099 a | N.D. a | N.D. a | N.D. a | C |
| | | 165°C | 0.193±0.074 a | 0.274±0.064 ab | 1.691±1.497 a | 0.248±0.122 a | |
| | | 190°C | 0.402±0.171 a | 0.479±0.354 b | 0.516±0.459 a | 0.466±0.490 a | |
| 1,879 | 5-hydroxyoctan-4-one | 140°C | N.D. a | 0.034±0.021 b | N.D. a | N.D. a | C |
| | | 165°C | N.D. a | N.D. a | N.D. a | N.D. a | |
| | | 190°C | N.D. a | N.D. a | N.D. a | N.D. a | |
| ***Lactones*** | | | | | | | |
| 805 | 2-methyloxolan-3-one | 140°C | N.D. a | N.D. a | N.D. a | N.D. a | A |
| | | 165°C | N.D. a | N.D. a | 0.394±0.115 a | 0.102±0.001 a | |
| | | 190°C | 1.041±0.171 b | 4.905±1.028 b | 2.464±0.940 b | 0.983±0.376 b | |
| 944 | 5-methyloxolan-2-one | 140°C | N.D. a | N.D. a | N.D. a | N.D. a | A |
| | | 165°C | N.D. a | N.D. a | N.D. a | N.D. a | |
| | | 190°C | N.D. a | 0.398±0.265 b | N.D. a | N.D. a | |
| 1,044 | 5-ethyloxolan-2-one | 140°C | N.D. a | N.D. a | N.D. a | N.D. a | A |
| | | 165°C | 0.699±0.318 b | 1.042±0.206 c | N.D. a | N.D. a | |
| | | 190°C | N.D. a | 0.558±0.284 b | 0.373±0.190 b | 0.099±0.030 b | |
| 1,248 | 5-butyloxolan-2-one | 140°C | N.D. a | N.D. a | N.D. a | N.D. a | A |
| | | 165°C | N.D. a | N.D. a | N.D. a | N.D. a | |
| | | 190°C | N.D. a | N.D. a | 0.085±0.080 a | N.D. a | |

**Table S1.** (Continued)

| RI[1)] | Volatile compounds | Heating temp. | Relative peak area[2)] Frying oil Soybean oil | Corn oil | Canola oil | Palm oil | ID[3)] |
|---|---|---|---|---|---|---|---|
| ***Lactones*** | | | | | | | |
| | | 140°C | N.D. a | N.D. a | N.D. a | N.D. a | |
| 805 | 2-methyloxolan-3-one | 165°C | N.D. a | N.D. a | 0.394±0.115 a | 0.102±0.001 a | A |
| | | 190°C | 1.041±0.171 b | 4.905±1.028 b | 2.464±0.940 b | 0.983±0.376 b | |
| | | 140°C | N.D. a | N.D. a | N.D. a | N.D. a | |
| 944 | 5-methyloxolan-2-one | 165°C | N.D. a | N.D. a | N.D. a | N.D. a | A |
| | | 190°C | N.D. a | 0.398±0.265 b | N.D. a | N.D. a | |
| | | 140°C | N.D. a | N.D. a | N.D. a | N.D. a | |
| 1,044 | 5-ethyloxolan-2-one | 165°C | 0.699±0.318 b | 1.042±0.206 c | N.D. a | N.D. a | A |
| | | 190°C | N.D. a | 0.558±0.284 b | 0.373±0.190 b | 0.099±0.030 b | |
| | | 140°C | N.D. a | N.D. a | N.D. a | N.D. a | |
| 1,248 | 5-butyloxolan-2-one | 165°C | N.D. a | N.D. a | N.D. a | N.D. a | A |
| | | 190°C | N.D. a | N.D. a | 0.085±0.080 a | N.D. a | |
| | | 140°C | N.D. a | 0.049±0.024 b | N.D. a | N.D. a | |
| 1,394 | 3-hydroxy-4,4-dimethyloxolan-2-one | 165°C | N.D. a | N.D. a | N.D. a | N.D. a | C |
| | | 190°C | N.D. a | N.D. a | N.D. a | N.D. a | |
| ***N-containing compounds*** | | | | | | | |
| | | 140°C | N.D. a | N.D. a | N.D. a | N.D. a | |
| 737 | pyridine | 165°C | 0.585±0.198 b | 0.774±0.263 b | 0.905±0.151 b | 0.218±0.047 a | A |
| | | 190°C | 0.842±0.002 c | 2.236±0.222 c | 1.150±0.551 b | 0.636±0.193 b | |
| | | 140°C | 3.302±1.313 a | 0.626±0.321 a | 1.492±0.509 a | 0.589±0.204 a | |
| 914 | methyl N-hydroxybenzenecarboximidate | 165°C | 1.319±1.355 a | 0.963±0.900 a | 1.193±0.330 ab | 2.160±0.443 b | C |
| | | 190°C | 2.666±0.790 a | 4.553±0.474 b | 2.420±0.569 b | 1.070±0.452 a | |

**Table S1.** (Continued)

| RI[1)] | Volatile compounds | Heating temp. | Relative peak area[2)] | | | | ID[3)] |
|---|---|---|---|---|---|---|---|
| | | | Frying oil | | | | |
| | | | Soybean oil | Corn oil | Canola oil | Palm oil | |
| ***N-containing compounds*** | | | | | | | |
| 737 | pyridine | 140°C | N.D. a | N.D. a | N.D. a | N.D. a | A |
| | | 165°C | 0.585±0.198 b | 0.774±0.263 b | 0.905±0.151 b | 0.218±0.047 a | |
| | | 190°C | 0.842±0.002 c | 2.236±0.222 c | 1.150±0.551 b | 0.636±0.193 b | |
| 914 | methyl N-hydroxybenzenecarboximidate | 140°C | 3.302±1.313 a | 0.626±0.321 a | 1.492±0.509 a | 0.589±0.204 a | C |
| | | 165°C | 1.319±1.355 a | 0.963±0.900 a | 1.193±0.330 ab | 2.160±0.443 b | |
| | | 190°C | 2.666±0.790 a | 4.553±0.474 b | 2.420±0.569 b | 1.070±0.452 a | |
| 1,012 | 1H-pyrrole-2-carbaldehyde | 140°C | N.D. a | N.D. a | N.D. a | N.D. a | A |
| | | 165°C | N.D. a | N.D. a | 0.315±0.113 b | 0.185±0.072 a | |
| | | 190°C | 2.256±0.394 b | 5.167±2.046 b | 4.021±0.236 c | 2.343±0.717 b | |
| 1,055 | 2-amino-1,4-dihydroimidazol-5-one | 140°C | N.D. a | N.D. a | N.D. a | N.D. a | C |
| | | 165°C | N.D. a | N.D. a | 0.241±0.063 b | N.D. a | |
| | | 190°C | N.D. a | 1.780±0.465 b | 0.688±0.140 c | N.D. a | |
| 1,060 | 1-(1H-pyrrol-2-yl)ethanone | 140°C | N.D. a | N.D. a | N.D. a | N.D. a | A |
| | | 165°C | N.D. a | N.D. a | N.D. a | N.D. a | |
| | | 190°C | N.D. a | N.D. a | 0.215±0.007 b | 0.088±0.037 b | |
| 1,109 | 2,2,6,6-tetramethylpiperidin-4-one;hydrate | 140°C | N.D. a | 0.905±0.356 b | 1.439±1.054 b | N.D. a | C |
| | | 165°C | N.D. a | N.D. a | N.D. a | N.D. a | |
| | | 190°C | N.D. a | N.D. a | N.D. a | N.D. a | |
| ***Phenols*** | | | | | | | |
| 1,048 | 2-methoxyphenol (guaiacol) | 140°C | N.D. a | N.D. a | N.D. a | N.D. a | A |
| | | 165°C | N.D. a | N.D. a | N.D. a | N.D. a | |
| | | 190°C | 0.156±0.021 b | 0.382±0.140 b | 0.240±0.061 b | 0.075±0.035 b | |

**Table S1.** (Continued)

| RI[1)] | Volatile compounds | Heating temp. | Relative peak area[2)] | | | | ID[3)] |
|---|---|---|---|---|---|---|---|
| | | | Frying oil | | | | |
| | | | Soybean oil | Corn oil | Canola oil | Palm oil | |
| ***Phenols*** | | | | | | | |
| 1,048 | 2-methoxyphenol (guaiacol) | 140°C | N.D. a | N.D. a | N.D. a | N.D. a | A |
| | | 165°C | N.D. a | N.D. a | N.D. a | N.D. a | |
| | | 190°C | 0.156±0.021 b | 0.382±0.140 b | 0.240±0.061 b | 0.075±0.035 b | |
| 1,264 | 4-ethyl-2-methoxyphenol (4-ethylguaiacol) | 140°C | N.D. a | N.D. a | N.D. a | N.D. a | B |
| | | 165°C | N.D. a | N.D. a | N.D. a | N.D. a | |
| | | 190°C | 0.179±0.062 b | 0.269±0.098 b | 0.252±0.064 b | 0.111±0.036 b | |
| 1,301 | 4-ethenyl-2-methoxyphenol | 140°C | N.D. a | N.D. a | N.D. a | N.D. a | B |
| | | 165°C | N.D. a | 0.130±0.014 a | 0.191±0.004 b | 0.125±0.035 b | |
| | | 190°C | 0.294±0.064 b | 0.407±0.125 b | 0.467±0.103 c | 0.215±0.059 c | |
| ***Pyrazines*** | | | | | | | |
| 726 | pyrazine | 140°C | N.D. a | N.D. a | N.D. a | N.D. a | A |
| | | 165°C | N.D. a | N.D. a | N.D. a | N.D. a | |
| | | 190°C | N.D. a | 0.367±0.216 b | 0.196±0.035 b | 0.147±0.062 b | |
| 818 | 2-methylpyrazine | 140°C | N.D. a | N.D. a | N.D. a | N.D. a | A |
| | | 165°C | 1.429±1.257 a | 0.953±0.551 a | 0.608±0.416 a | 0.293±0.190 a | |
| | | 190°C | 1.827±1.529 a | 6.072±2.476 b | 3.426±0.464 b | 2.482±0.853 b | |
| 907 | 2,6-dimethylpyrazine | 140°C | N.D. a | N.D. a | N.D. a | N.D. a | A |
| | | 165°C | 1.615±1.148 b | 1.406±0.812 a | 0.945±0.392 b | 0.453±0.148 a | |
| | | 190°C | 1.737±0.645 b | 7.080±3.678 b | 3.276±0.507 c | 2.122±0.659 b | |
| 910 | 2-ethylpyrazine | 140°C | N.D. a | N.D. a | N.D. a | N.D. a | A |
| | | 165°C | N.D. a | N.D. a | N.D. a | N.D. a | |
| | | 190°C | N.D. a | 0.418±0.390 a | 0.249±0.110 b | 0.123±0.025 b | |
| 912 | 2,3-dimethylpyrazine | 140°C | N.D. a | N.D. a | N.D. a | N.D. a | B |
| | | 165°C | N.D. a | N.D. a | N.D. a | N.D. a | |
| | | 190°C | N.D. a | 0.241±0.191 b | N.D. a | N.D. a | |

**Table S1.** (Continued)

| RI[1)] | Volatile compounds | Heating temp. | Relative peak area[2)] | | | | ID[3)] |
|---|---|---|---|---|---|---|---|
| | | | Frying oil | | | | |
| | | | Soybean oil | Corn oil | Canola oil | Palm oil | |
| ***Pyrazines*** | | | | | | | |
| 995 | 2,3,5-trimethylpyrazine | 140°C | N.D. a | N.D. a | N.D. a | N.D. a | B |
| | | 165°C | N.D. a | N.D. a | 0.133±0.032 b | N.D. a | |
| | | 190°C | N.D. a | 0.601±0.398 b | 0.424±0.076 c | 0.150±0.063 b | |
| 1,010 | 2-ethenyl-5-methylpyrazine | 140°C | N.D. a | N.D. a | N.D. a | N.D. a | B |
| | | 165°C | N.D. a | N.D. a | N.D. a | N.D. a | |
| | | 190°C | N.D. a | 0.356±0.259 b | 0.315±0.094 b | 0.104±0.045 b | |
| ***S-containing compounds*** | | | | | | | |
| <600 | methylsulfanylmethane (dimethyl sulfide) | 140°C | 0.563±0.174 a | 0.218±0.107 a | 0.346±0.160 a | 0.176±0.110 a | B |
| | | 165°C | 0.705±0.390 a | 0.944±0.215 b | 0.775±0.105 b | 0.824±0.200 c | |
| | | 190°C | 0.595±0.196 a | 1.116±0.392 b | 1.098±0.297 b | 0.467±0.098 b | |
| 608 | propane-1-thiol | 140°C | 1.219±0.186 ab | 0.432±0.321 a | 0.931±0.495 a | 0.226±0.136 a | A |
| | | 165°C | 1.786±0.515 b | 1.998±0.278 b | 3.551±1.009 b | 2.220±1.275 b | |
| | | 190°C | 0.722±0.541 a | 2.632±1.128 b | 4.626±1.842 b | 1.114±0.344 ab | |
| 611 | 2-methylthiirane | 140°C | 0.240±0.041 b | 0.086±0.054 a | N.D. a | 0.097±0.050 a | B |
| | | 165°C | N.D. a | N.D. a | N.D. a | 0.462±0.129 b | |
| | | 190°C | N.D. a | 0.255±0.067 b | 0.293±0.105 b | 0.152±0.146 a | |
| 612 | prop-2-ene-1-thiol | 140°C | 0.141±0.033 b | 0.058±0.034 b | N.D. a | 0.039±0.015 a | A |
| | | 165°C | 0.290±0.082 c | N.D. a | N.D. a | 0.108±0.094 a | |
| | | 190°C | N.D. a | N.D. a | N.D. a | N.D. a | |
| 771 | 3-methylthiophene | 140°C | N.D. a | N.D. a | N.D. a | N.D. a | A |
| | | 165°C | N.D. a | N.D. a | N.D. a | N.D. a | |
| | | 190°C | N.D. a | 0.279±0.073 b | 0.289±0.017 b | 0.149±0.046 b | |
| 734 | (methyldisulfanyl)methane (dimethyl disulfide) | 140°C | 0.296±0.038 a | 0.101±0.102 a | 0.193±0.072 a | 0.101±0.018 a | A |
| | | 165°C | 2.121±1.219 a | 2.905±1.185 a | 5.948±0.734 b | 2.586±1.134 b | |
| | | 190°C | 4.509±1.041 b | 17.943±3.296 b | 10.471±2.189 c | 5.259±1.313 c | |

**Table S1.** (Continued)

| RI[1)] | Volatile compounds | Heating temp. | Relative peak area[2)] | | | | ID[3)] |
|---|---|---|---|---|---|---|---|
| | | | Frying oil | | | | |
| | | | Soybean oil | Corn oil | Canola oil | Palm oil | |
| ***S-containing compounds*** | | | | | | | |
| 752 | 1-methylsulfanylpropane (methyl propyl sulfide) | 140°C | N.D. a | N.D. a | N.D. a | N.D. a | B |
| | | 165°C | 0.317±0.167 b | 0.385±0.148 b | 0.460±0.137 b | 0.280±0.131 b | |
| | | 190°C | 0.332±0.092 b | 1.330±0.031 c | 0.681±0.158 b | 0.358±0.041 b | |
| 870 | 3,4-dimethylthiophene | 140°C | 0.133±0.031 b | 0.050±0.024 a | 0.069±0.034 a | 0.041±0.013 a | B |
| | | 165°C | N.D. a | N.D. a | 0.205±0.038 b | 0.283±0.082 b | |
| | | 190°C | N.D. a | 0.197±0.040 b | 0.164±0.023 b | 0.099±0.017 a | |
| 898 | 2,5-dimethylthiophene | 140°C | 0.761±0.061 a | 0.312±0.232 a | 0.403±0.176 a | 0.241±0.074 a | A |
| | | 165°C | 1.638±0.547 b | 2.287±0.236 b | 2.630±0.532 b | 2.904±0.762 b | |
| | | 190°C | 1.419±0.098 b | 5.431±1.055 c | 3.836±0.122 c | 2.154±0.511 b | |
| 904 | 3-methylsulfanylpropanal (methional) | 140°C | N.D. a | 0.032±0.021 b | N.D. a | N.D. a | A |
| | | 165°C | 0.513±0.117 b | N.D. a | N.D. a | N.D. a | |
| | | 190°C | N.D. a | N.D. a | N.D. a | N.D. a | |
| 915 | 2-methoxythiophene | 140°C | N.D. a | 0.095±0.042 b | N.D. a | 0.101±0.046 b | B |
| | | 165°C | N.D. a | N.D. a | N.D. a | N.D. a | |
| | | 190°C | N.D. a | N.D. a | N.D. a | N.D. a | |
| 919 | 3-prop-2-enylsulfanylprop-1-ene (diallyl sulfide) | 140°C | N.D. a | 0.035±0.022 b | N.D. a | N.D. a | B |
| | | 165°C | N.D. a | N.D. a | 0.197±0.058 a | 0.125±0.038 b | |
| | | 190°C | N.D. a | N.D. a | N.D. b | N.D. a | |
| 922 | 1-(methyldisulfanyl)prop-1-ene (methyl (Z)-1-propenyl disulfide) | 140°C | 0.440±0.043 b | 0.076±0.024 a | 0.184±0.081 a | 0.099±0.024 a | B |
| | | 165°C | 0.570±0.159 b | 0.765±0.195 b | 0.665±0.194 b | 0.367±0.147 b | |
| | | 190°C | N.D. a | 0.905±0.313 b | 1.242±0.349 c | 0.408±0.177 b | |
| 924 | 1-(methyldisulfanyl)propane (methyl propyl disulfide) | 140°C | 1.359±0.413 a | 0.294±0.165 a | 0.805±0.360 a | 0.308±0.072 a | A |
| | | 165°C | 3.032±1.454 a | 3.183±1.018 a | 8.648±4.803 ab | 3.393±0.929 b | |
| | | 190°C | 3.009±0.743 a | 11.611±3.281 b | 12.987±5.997 b | 4.803±1.625 b | |

**Table S1.** (Continued)

| RI[1)] | Volatile compounds | Heating temp. | Relative peak area[2)] | | | | ID[3)] |
|---|---|---|---|---|---|---|---|
| | | | Frying oil | | | | |
| | | | Soybean oil | Corn oil | Canola oil | Palm oil | |
| ***S-containing compounds*** | | | | | | | |
| 930 | 1-(methyldisulfanyl)prop-1-ene (methyl (E)-1-propenyl disulfide) | 140°C | 0.715±0.159 b | 0.164±0.050 a | 0.332±0.160 a | 0.201±0.058 a | B |
| | | 165°C | 0.917±0.285 b | 1.065±0.302 b | 0.909±0.130 ab | 1.019±0.412 b | |
| | | 190°C | 0.288±0.114 a | 1.979±0.235 c | 1.411±0.791 b | 0.423±0.172 a | |
| 936 | 1-propylsulfanylpropane (dipropyl sulfide) | 140°C | N.D. a | N.D. a | N.D. a | N.D. a | B |
| | | 165°C | 0.396±0.229 b | 0.877±0.351 a | 0.626±0.242 b | 0.378±0.080 b | |
| | | 190°C | 0.773±0.177 c | 2.452±0.775 b | 1.335±0.181 c | 0.650±0.233 b | |
| 950 | 1-ethylsulfanyl-2-methylprop-1-ene | 140°C | N.D. a | N.D. a | N.D. a | N.D. a | C |
| | | 165°C | 0.545±0.290 b | 0.475±0.140 b | 0.422±0.135 b | 0.303±0.115 b | |
| | | 190°C | N.D. a | 0.428±0.120 b | 0.327±0.113 b | 0.122±0.032 a | |
| 957 | (methyltrisulfanyl)methane (dimethyl trisulfide) | 140°C | 0.986±0.132 a | 0.271±0.262 a | 0.486±0.209 a | 0.300±0.049 a | A |
| | | 165°C | 6.989±3.363 b | 8.732±4.172 a | 15.658±2.301 b | 6.743±1.965 b | |
| | | 190°C | 7.375±1.220 b | 22.589±6.108 b | 13.443±3.122 b | 6.581±2.693 b | |
| 964 | 2-ethylthiophene | 140°C | N.D. a | N.D. a | N.D. a | N.D. a | C |
| | | 165°C | N.D. a | N.D. a | N.D. a | N.D. a | |
| | | 190°C | 0.163±0.038 b | 0.327±0.173 b | 0.409±0.098 b | 0.198±0.101 b | |
| 1,073 | 2-methyl-2-(methyldisulfanyl)propane (methyl tert-butyl disulfide) | 140°C | N.D. a | 0.079±0.039 a | N.D. a | N.D. a | C |
| | | 165°C | 0.444±0.109 b | 0.859±0.111 b | 0.564±0.194 b | 0.405±0.179 b | |
| | | 190°C | N.D. a | N.D. a | N.D. a | N.D. a | |
| 1,100 | 1-(propyldisulfanyl)propane (dipropyl disulfide) | 140°C | 5.114±0.952 ab | 0.876±0.056 a | 2.014±0.884 a | 0.892±0.230 a | B |
| | | 165°C | 8.311±2.641 b | 8.151±1.504 b | 7.473±3.200 ab | 2.829±0.589 ab | |
| | | 190°C | 2.030±0.607 a | 6.001±2.202 b | 12.553±6.475 b | 3.651±1.920 b | |
| 1,108 | 1-(prop-2-enyldisulfanyl)propane (allyl propyl disulfide) | 140°C | 2.823±0.344 b | 0.395±0.115 a | 0.939±0.404 a | 0.509±0.180 a | B |
| | | 165°C | 3.771±0.483 c | 4.266±1.274 b | 2.272±0.371 b | 1.338±0.325 b | |
| | | 190°C | 0.998±0.282 a | 2.980±0.837 b | 3.221±0.950 b | 1.047±0.512 ab | |

**Table S1.** (Continued)

| RI[1)] | Volatile compounds | Heating temp. | Relative peak area[2)] | | | | ID[3)] |
|---|---|---|---|---|---|---|---|
| | | | Frying oil | | | | |
| | | | Soybean oil | Corn oil | Canola oil | Palm oil | |
| ***S-containing compounds*** | | | | | | | |
| 1,199 | (methyltetrasulfanyl)methane (dimethyl tetrasulfide) | 140°C | 0.157±0.089 a | 0.024±0.020 a | 0.024±0.042 a | 0.029±0.007 a | B |
| | | 165°C | 0.482±0.138 a | 0.749±0.251 a | 3.033±2.065 b | 0.562±0.246 a | |
| | | 190°C | 1.561±0.580 b | 1.550±1.225 b | 1.278±1.197 ab | 0.954±0.939 a | |
| 1,298 | 1-(propyldisulfanyl)pentane (pentyl propyl disulfide) | 140°C | N.D. a | N.D. a | N.D. a | N.D. a | C |
| | | 165°C | N.D. a | N.D. a | N.D. a | N.D. a | |
| | | 190°C | N.D. a | 0.250±0.131 b | 0.175±0.123 b | 0.045±0.026 b | |
| 1,316 | 1-(propyltrisulfanyl)propane (dipropyl trisulfide) | 140°C | 0.803±0.591 a | 0.083±0.034 a | 0.177±0.073 a | 0.080±0.024 a | A |
| | | 165°C | 1.026±0.117 a | 1.233±0.451 b | 3.326±3.410 a | 0.296±0.069 a | |
| | | 190°C | 0.634±0.246 a | 1.604±0.674 b | 3.254±1.893 a | 1.434±1.226 a | |
| 1,330 | (3R,5R)-3,5-diethyl-1,2,4-trithiolane | 140°C | 0.543±0.218 a | 0.097±0.042 a | 0.110±0.030 a | 0.089±0.027 a | B |
| | | 165°C | 0.389±0.210 a | 0.452±0.123 b | 0.519±0.162 b | 0.375±0.122 b | |
| | | 190°C | 0.359±0.156 a | 0.793±0.201 c | 0.450±0.127 b | 0.220±0.060 ab | |
| 1,353 | 5-methyltetrathiane | 140°C | N.D. a | N.D. a | N.D. a | N.D. a | B |
| | | 165°C | N.D. a | N.D. a | N.D. a | N.D. a | |
| | | 190°C | N.D. a | 0.189±0.082 b | N.D. a | 0.051±0.026 b | |
| 1,631 | 1-decylsulfanyldecane (decyl sulfide) | 140°C | 0.133±0.050 b | 0.082±0.069 b | N.D. a | 0.028±0.009 b | C |
| | | 165°C | N.D. a | N.D. a | 0.158±0.049 b | N.D. a | |
| | | 190°C | N.D. a | N.D. a | N.D. a | N.D. a | |
| ***N and S-containing compounds*** | | | | | | | |
| 1,083 | 2-methylsulfanylpyridine | 140°C | N.D. a | N.D. a | N.D. a | N.D. a | A |
| | | 165°C | N.D. a | N.D. a | N.D. a | N.D. a | |
| | | 190°C | N.D. a | 0.441±0.150 b | 0.309±0.038 b | 0.192±0.066 b | |

**Table S1.** (Continued)

| RI[1)] | Volatile compounds | Heating temp. | Relative peak area[2)] | | | | ID[3)] |
|---|---|---|---|---|---|---|---|
| | | | Frying oil | | | | |
| | | | Soybean oil | Corn oil | Canola oil | Palm oil | |
| ***Others*** | | | | | | | |
| 760 | thietane 1-oxide | 140°C | 1.346±0.302 b | 0.215±0.063 b | 0.403±0.165 b | 0.135±0.078 b | C |
| | | 165°C | N.D. a | N.D. a | N.D. a | N.D. a | |
| | | 190°C | N.D. a | N.D. a | N.D. a | N.D. a | |
| 1,077 | 2-methyl-3-methylsulfanylfuran | 140°C | N.D. a | N.D. a | N.D. a | N.D. a | C |
| | | 165°C | N.D. a | N.D. a | N.D. a | N.D. a | |
| | | 190°C | 0.348±0.136 b | 0.473±0.223 b | 0.572±0.201 b | 0.192±0.075 b | |
| 1,213 | 2-methyl-5-methylsulfanylfuran | 140°C | N.D. a | N.D. a | N.D. a | N.D. a | C |
| | | 165°C | N.D. a | N.D. a | 0.175±0.069 b | 0.131±0.017 b | |
| | | 190°C | 0.260±0.046 b | 0.437±0.159 b | 0.295±0.043 c | 0.157±0.036 b | |

1) Retention indices were calculated using n-alkanes $C_7$-$C_{30}$ as external standards.
2) Mean values of relative peak area to that of internal standard ± standard deviation (n=3).
3) Identification of the compounds was based as follows: A, mass spectrum and retention index agreed with those of authentic compounds under the same conditions (positive identification); B, mass spectrum and retention index were consistent with those from NIST database (tentative identification); C, mass spectrum was consistent with that of W9N08 (Wiley and NIST) and manual interpretation (tentative identification).
4) Not detected.
5) Different letters means significant differences ($p$<0.05) between samples using Duncan's multiple comparison test.
